# Barnyard Millet for Food and Nutritional Security: Current Status and Future Research Direction

**DOI:** 10.3389/fgene.2020.00500

**Published:** 2020-06-23

**Authors:** Vellaichamy Gandhimeyyan Renganathan, Chockalingam Vanniarajan, Adhimoolam Karthikeyan, Jegadeesan Ramalingam

**Affiliations:** ^1^Department of Plant Breeding and Genetics, Agricultural College & Research Institute, Tamil Nadu Agricultural University, Madurai, India; ^2^Department of Biotechnology, Centre of Innovation, Agricultural College & Research Institute, Tamil Nadu Agricultural University, Madurai, India

**Keywords:** barnyard millet, *Echinochloa* species, micronutrients, small millets, genetic and genomic resources, value addition

## Abstract

Barnyard millet (*Echinochloa* species) has become one of the most important minor millet crops in Asia, showing a firm upsurge in world production. The genus *Echinochloa* comprises of two major species, *Echinochloa esculenta* and *Echinochloa frumentacea*, which are predominantly cultivated for human consumption and livestock feed. They are less susceptible to biotic and abiotic stresses. Barnyard millet grain is a good source of protein, carbohydrate, fiber, and, most notably, contains more micronutrients (iron and zinc) than other major cereals. Despite its nutritional and agronomic benefits, barnyard millet has remained an underutilized crop. Over the past decades, very limited attempts have been made to study the features of this crop. Hence, more concerted research efforts are required to characterize germplasm resources, identify trait-specific donors, develop mapping population, and discover QTL/gene (s). The recent release of genome and transcriptome sequences of wild and cultivated *Echinochloa* species, respectively has facilitated in understanding the genetic architecture and decoding the rapport between genotype and phenotype of micronutrients and agronomic traits in this crop. In this review, we highlight the importance of barnyard millet in the current scenario and discuss the up-to-date status of genetic and genomics research and the research gaps to be worked upon by suggesting directions for future research to make barnyard millet a potential crop in contributing to food and nutritional security.

## Introduction

**FIGURE 1:**
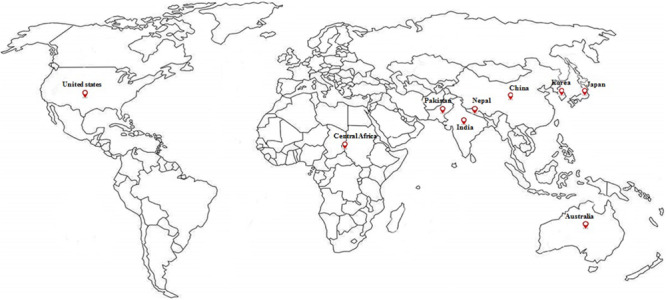
Major cultivation and production of barnyard millet around the world (from IIMR, 2018).

**FIGURE 2:**
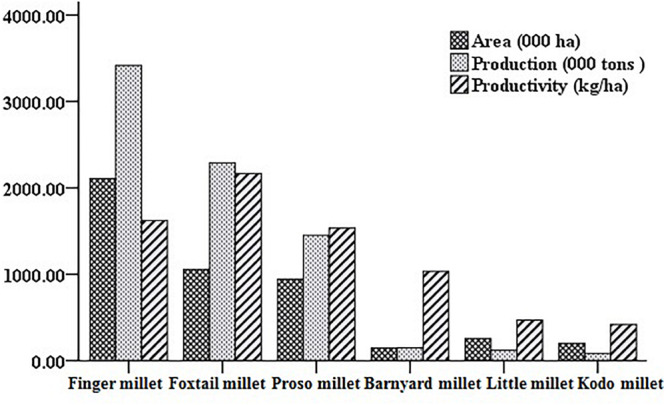
World area, production, and productivity scenario of small millets (from IIMR, 2018).

Barnyard millet (*Echinochloa* species) is an ancient millet crop grown in warm and temperate regions of the world and widely cultivated in Asia, particularly India, China, Japan, and Korea. It is the fourth most produced minor millet, providing food security to many poor people across the world. Globally, India is the biggest producer of barnyard millet, both in terms of area (0.146 m ha^–1^) and production (0.147 mt) with average productivity of 1034 kg/ha during the last 3 years (IIMR, 2018). The details on major areas of cultivation and worldwide production are presented in Figures 1, 2. Barnyard millet is primarily cultivated for human consumption, though it is also used as a livestock feed. Among many cultivated and wild species of barnyard millet, two of the most popular species are *Echinochloa frumentacea* (Indian barnyard millet) and *Echinochloa esculenta* (Japanese barnyard millet) (Sood et al., 2015). Barnyard millet is a short duration crop that can grow in adverse environmental conditions with almost no input and can withstand various biotic and abiotic stresses. In addition to these agronomic advantages, the grains are valued for their high nutritional value and lower expense as compared to major cereals like rice, wheat, and maize. It contains a rich source of protein, carbohydrates, fiber, and, most notably, micronutrients like iron (Fe) and zinc (Zn) (Singh et al., 2010; Saleh et al., 2013; Chandel et al., 2014) that are related to numerous health benefits (Saleh et al., 2013; Ugare et al., 2014). All these features make barnyard millet an ideal supplementary crop for subsistence farmers and also as an alternate crop during the failure of monsoons in rice/major crop cultivating areas (Gupta et al., 2009).

Despite barnyard millet’s excellent nutritional and agronomic value, the lack of awareness has led this crop to be considered as a neglected and underutilized crop. Over the past decades, efforts made to study the features of barnyard millet are limited compared to other minor millets. So far, most of the studies have been aimed at exploring the knowledge of diversity within the germplasm through morphological (Mehta et al., 2005; Gupta et al., 2009; Nirmalakumari and Vetriventhan, 2009; Sood et al., 2015; Renganathan et al., 2017) and molecular markers (Nozawa et al., 2006; Altop and Mennan, 2011; Prabha et al., 2012; Wallace et al., 2015; Manimekalai et al., 2018; Murukarthick et al., 2019). Also, several studies disclosed the nutritional profile of barnyard millet, particularly the high Fe and Zn content in the grains (Veena et al., 2005; Saleh et al., 2013; Chandel et al., 2014; Renganathan et al., 2017). However, comprehensive research is needed to understand the accurate details of germplasm accessions, identify the trait-specific donors, develop mapping population, and discover the quantitative trait locus (QTLs) and gene. Genomic resources are helpful for the progress of any crop species and they assist effective characterization of germplasm resources and their subsequent use in the discovery of QTL/gene(s) for the crop improvement program. However, genome research in barnyard millet is still in the early stage and far behind the other minor millets. This is mainly due to the complex nature of the genome (2*n* = 6*x* = 54, hexaploid). Recently, second and third-generation sequencing technologies unlocked several genome sequencing issues and facilitated to identify the genome sequence of wild and transcriptome sequences in cultivated *Echinochloa* species (Li et al., 2013a, b; Yang et al., 2013; Nah et al., 2015; Xu et al., 2015; Guo et al., 2017; Murukarthick et al., 2019). These genome resources facilitated the chance for better genotyping studies such as genetic diversity analysis, development of highly dense linkage maps and accurate physical maps, and detection of QTLs associated with micronutrients and agronomic traits. For instance, Wallace et al. (2015) developed the genome through single nucleotide polymorphism (SNP) markers and analyzed the genetic diversity in the barnyard millet core collection. Murukarthick et al. (2019) investigated the transcriptional changes between *E. frumentacea* and *E. cru-galli* and discovered genes related to drought and micronutrient content.

**FIGURE 3:**
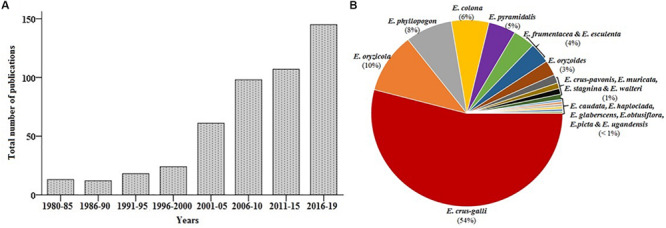
Graphical representations of literature and nucleotide sequence availability. **(A)** PubMed (https://www.ncbi.nlm.nih.gov/pubmed) **(B)** nucleotide sequence (https://www.ncbi.nlm.nih.gov/). (Note: Data verified till December 2019).

Accumulating evidence suggests that the interest in barnyard millet research has increased markedly over recent years; since 2010, more than 350 publications on barnyard millet have been available in the National Center for Biotechnology Information (NCBI) PubMed database^1^ (last accessed on December 2019) (Figure 3A). This review discusses the origin and taxonomy, nutritional value and health benefits, stress adaptation as well as the current status of genetic and genomics research in barnyard millet. The final section highlights the research gap and future research directions needed to promote barnyard millet as a potential crop for food and nutritional security.

## Origin, Taxonomy and Genomic Relationship of *Echinochloa* Species

Barnyard millet belongs to the genus *Echinochloa*, the family *Poaceae*, and the sub-family *Panicoideae* (Clayton and Renvoize, 2006). The genus *Echinochloa* consists of approximately 250 annual and perennial species that are widely distributed in the warmer and temperate parts of the world (Bajwa et al., 2015). However, the lack of clarity over the *Echinochloa* species makes it hard to differentiate themselves via the morphological markers due to low interspecific and intraspecific variations in nature and their phenotype plasticity (Chauhan and Johnson, 2011). Despite this challenge, 35 species have been identified to date for their taxa and phylogenetic relationship through morphological, cytological, and molecular marker studies (Yabuno, 1966, 1987; Yuichiro et al., 1999; Yamaguchi et al., 2005). Among them, most of the *Echinochloa* species, including *E. crus-galli* (allohexaploid, 2*n* = 6*x* = 54), *E. colona* (allohexaploid, 2*n* = 6*x* = 54), *E. oryzicola* (allotetraploid, 2*n* = 4*x* = 36), and others, have been designated as problematic weeds in major crop fields (Yabuno, 1966, 1987; Wanous, 1990; Yuichiro et al., 1999; Yamaguchi et al., 2005; Kraehmer et al., 2015). *E. crus-galli* is a predominant weed in rice fields in more than 60 countries, due to its quick germination (even in hypoxic conditions, up to 100 mm deep), rapid growth, mimicking character of rice, broad ecological tolerance, and profuse seed production (Barrett, 1983).

*Echinochloa* species have very few cultivatable forms and thereby are cultivated as minor millet by marginal farmers in warmer and temperate regions of the world. *E. frumentacea* (Roxb.) Link; syn. *E. colona* var. *frumentacea* (allohexaploid, 2*n* = 6*x* = 54), commonly known as Indian barnyard millet, originated from wild *E. colona* (L.) (Jungle rice), and exhibits a parallel line of evolution in India and Africa. *E. frumentacea* species has four races, namely *stolonifera*, *intermedia*, *robusta*, and *laxa*, that are widely cultivated in Central Africa, India, Malawi, Nepal, Pakistan, and Tanzania (Doggett, 1989; Upadhyaya et al., 2014). Another cultivated allohexaploid species, *E. esculenta* (A. Braun) H. Scholz; syn. *E. utilis* var. *esculenta*; known as Japanese barnyard millet, originated from wild *E. crus-galli* (L.) (Barnyard grass) was domesticated some 4,000 years ago in the temperate regions of Japan (De Wet et al., 1983; Doggett, 1989). *Utilis* and *intermedia* are two races of *E. crus-galli*, widely cultivated in Japan, Korea, China, Russia, and Germany (De Wet et al., 1983; Yabuno, 1987; Upadhyaya et al., 2014). Both wild and cultivated *Echinochloa* species are different from each other in terms of growth habitat, general morphology, and other characteristics (Table 1). The interspecific relationship between *Echinochloa* species was unclear till a series of prominent taxonomic reports by Yabuno, (1962, 1984, 1996, 2001). The interspecific hybrids between wild species and its progenitor, i.e., *E. crus-galli* × *E. esculenta* and *E. colona* × *E. frumentacea* produce normal meiotic division (27 bivalents) i.e., fertile. But, interspecific hybrids between two cultivated species and their respective wild counterparts, *E. esculenta* × *E. frumentacea* and *E. crus-galli* × *E. colona*, showed irregular meiotic division that leads to sterility (Yabuno, 1966, 1984). Collectively, all the cytological studies reveal the poor genomic affinity among species of *Echinochloa*. Besides, Yamaguchi et al. (2005) confirmed three cross compatible groups identified by Yabuno (1966) using chloroplast DNA sequence analysis, and separated these *Echinochloa* complexes into *E. oryzicola* complex, *E. crus-galli* complex, and *E. colona-frumentacea* complex. The *E. oryzicola* complex consists of two weedy species, *E. oryzicola* and *E. phyllopogon*, and one rare cultivated Mosuo barnyard millet. The *E. crus-galli* complex includes four wild species, *E. crus-galli* var. *crus-galli*, *E. crus-galli* var. *praticola*, *E. crus-galli* var. *formosensis*, and *E. crus-galli* var. *oryzoides*, and one major cultivated species, *E. esculenta* (Japanese barnyard millet). The third, *E. colona-frumentacea* complex, consists of one wild species, *E. colona*, and one major cultivated species, *E. frumentacea* (Indian barnyard millet). Further, through molecular analysis, Aoki and Yamaguchi (2008) reported that, though all these three groups exhibit different cytoplasmic lineages, the nuclear lineage between *E. oryzicola* complex and *E. crus-galli* complex have a higher affinity than *E. colona-frumentacea* complexes proving Yabuno’s hypothesis that *E. oryzicola* is the probable paternal parent of *E. crus-galli* (Aoki and Yamaguchi, 2008). However, information regarding ancestors of *E. colona* and their cultivated *E. frumentacea* remains almost unknown. Therefore, analyses the meiotic behavior of inter or intra specific hybrid combinations through advanced cytogenetic techniques like genomic in situ hybridization (GISH)/fluorescent in situ hybridization (FISH) would not only provides their ancestral information but also differentiate many unresolved genomes of *Echinochloa* species.

**TABLE 1:** Morphological differences among wild and cultivated species of *Echinochloa* species.

**Traits**	***Echinochloa colona^a^***	***Echinochloa crus-galli^a^***	***Echinochloa frumentacea***	***Echinochloa esculenta***
Common name	Jungle rice	Barnyard grass	Indian barnyard millet/sawa millet	Japanese barnyard millet
Synonyms	*Echinochloa colonum*, *Echinochloa crus-galli* subsp. *colona*, *Panicum colonum*, *Panicum cumingianum*, *Panicum zonale*, and *Milium colonum*, *Oplismenus colonus*	*Panicum crus-galli*, *Panicum hispidulum*, *Milium crus-galli*, and *Pennisetum crus-galli*	Billion Dollar grass, sawa millet, sama millet	Japanese millet, marsh millet, Siberian millet, and white millet
Origin	China and Japan	China, Japan, and Korea	India, Pakistan, and Nepal	Eastern Asia, Japan, China, and Korea
Distribution	Widely distributed in South and Southeast Asia, Australia, Africa, Europe, and America	Widely distributed in South and Southeast Asia, Africa, Europe, and America	Widely distributed in Central Africa, Africa, Temperate Asia, Tropical Asia, Australasia, and South America.	Widely distributed in India, Temperate Asia, Australasia, and Pacific.
Habitat	Annual, warmer region	Annual, temperate region	Annual, warmer region	Annual, warm-season
Cultivable range	–	–	Altitude below 1,900 m	Altitude up to 2,500 m
General morphology	Erect to decumbent, 60 cm tall, short leaf length 10-15 cm, red tinges at the basal portion of leaf, leaf blade surface smooth, leaf blades 3–30 cm long and 2–8 mm wide	Erect, 200 cm tall, leaf 10-40 cm long, leaf blade surface smooth, leaf blades 0.5–35 cm long and 6–20 mm wide	Erect, 242 cm tall, leaf length 15–40 cm long and 1–2.5 cm wide, plants mostly green, however, purple tinges also found in vegetative and reproductive parts, leaf blades are smooth and glabrous, culms slender to robust	Robust, 60–122 cm tall, leaf sheath smooth 10–50 cm long and 7–25 mm wide, plants green, however, light to dark purple pigmentation in various plant parts, thicker stem
Inflorescence morphology	Green to purple, inflorescence length 5-15 cm, simple ascending racemes 5–15 numbers, 0.5–3 cm long raceme	Green to purple, inflorescence length 10-25 cm, compound ascending racemes 5–15 numbers, 2–10 cm long, slightly hairy, green to purplish awns 2-5 mm long	Green to purple, usually erect and compact, inflorescence length 1-28 cm long, racemes numerous 20–70, 1–3 cm long, rarely drooping, awnless	Brown to purple, compact inflorescence 12–15 cm long, racemes 5–15 numbers, arcuate to flexuous, 0.5–3 cm long, rarely awned
Spikelets on panicle	Spikelets arranged in 4 uniform rows on the primary rachis, spikelets 1-3 mm long	Open, branched spikelets on the rachis, setae on the primary branches	Compact, non-branched spikelets on the rachis, tightly clustered, 2–4 mm long, acute and awnless.	Compact, branched spikelets, larger spikelets and longer primary branches, dense clustered, 3–4 mm long, shortly cuspidate and rarely awned.
Caryopsis	Straw white, brown	Dark gray, brown	Turgid, whitish	Dull, pale yellow to light brown
Seed dormancy	Present	Absent	Present	Absent
Seed shattering	High	Low	High	Low

**^a^http://www.knowledgebank.irri.org/training/fact-sheets/item/echinochloa. Source: Yabuno, 1987; Muldoon et al., 1982; De Wet et al., 1983; Doggett, 1989; Bandyopadhyay, 1999*; *Yadav and Yadav, 2013; Renganathan et al., 2015.**

## Plant Architecture, Floral Biology and Seed Traits

**FIGURE 4:**
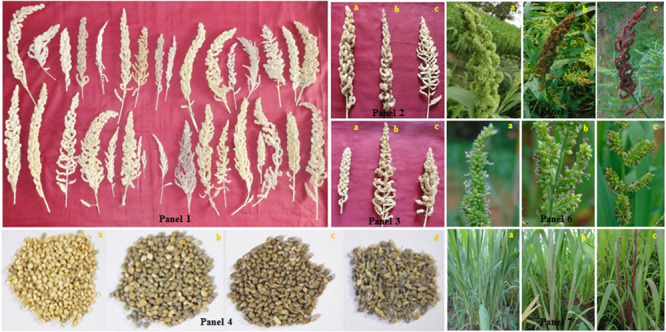
Genetic diversity of various morphological traits of barnyard millet. **(Panel 1)** Ear head diversity of different barnyard millet accessions. **(Panel 2)** Variation in ear head compactness **(a)** compact, **(b)** intermediate, and **(c)** open. **(Panel 3)** Variation in ear head shape **(a)** cylindrical, **(b)** pyramidal, and **(c)** globose to elliptic. **(Panel 4)** Grain variation in color and size of different barnyard millet accessions **(a)** straw white, medium **(b)** light gray, bold **(c)** gray, medium, and **(d)** dark gray, narrow. **(Panel 5)** variation in ear head color **(a)** green, **(b)** medium purple, and **(c)** dark purple. **(Panel 6)** Anther color variation **(a)** white, **(b)** light purple, and **(c)** dark purple. **(Panel 7)** Variation in tillering ability and pigmentation **(a)** high tillers, green, **(b)** medium tillers, light pigmentation, and **(c)** low tillers, dark pigmentation (from Prakash and Vanniarajan, 2013; Renganathan et al., 2017; Kuraloviya et al., 2019).

The cultivated barnyard millet is an annual, robust, and tall crop that grows up to a height of 220 cm (Denton, 1987; Padulosi et al., 2009). The inflorescence is a terminal panicle with varying shapes (cylindrical, pyramidal, and globose to elliptic), colors (green, light purple, and dark purple) and compactness (compact, intermediate, and open) (Gupta et al., 2009; Prakash and Vanniarajan, 2013; Sood et al., 2014; Renganathan et al., 2017; Kuraloviya et al., 2019) (Figure 4). Racemes are present in one side, two sides, or around the axis of rachis and vary from 22 to 64 numbers per inflorescence (Renganathan et al., 2017). The arrangement of spikelets is either on one side or around the rachis of the raceme. Each spikelet contains two florets in which the lower floret is sterile and consists of lemma with small palea, while the upper floret is bisexual with a shiny lemma that partially encloses palea. Fertile lemma and palea have three stamens varying from a white color to a dark purplish color with stigma plumose and bifid, ranging from white to dark purple (Figure 4). The two unequal glumes further enclose the seed kernel (Gupta et al., 2010b; Singh et al., 2010). Anthesis and pollination progress in the direction from top to bottom of the inflorescence in the early morning (5 am) and reaches a maximum during 6 am -7 am, while it closes at 10 am (Sundararaj and Thulasidas, 1976; Jayaraman et al., 1997). Sundararaj and Thulasidas (1976) further reported on the requirement of 10–14 days of duration for the flowering process. Though self-pollination is a strict rule, the reception of stigmatic branches before dehiscence of anther provides some chances for cross-pollination (Seetharam et al., 2003).

Compared to other minor millets like kodo and foxtail millet, barnyard millet grains are less hard. The mature pericarp of the seed consists of two epidermal layers with cells of the inner epidermis completely compressed over the outer epidermis (Singh et al., 2010). The cell wall of the aleuronic layer cutinized (Zee and O’brien, 1971), and also contains a maximum amount of carbohydrate (57–66%), followed by fiber (6.4–12.2%), protein (5–8.5%), fat (3.5–4.6%), and ash (2.5–4.0%) content. Starch granules are simple and are spherical to polygonal shapes with a diameter of 1.2–10 μm, which is larger than other small millets (Kumari and Thayumanavan, 1998). The pericarp color of grain differs among genotypes from straw white to light gray and dark gray (Renganathan et al., 2017; Kuraloviya et al., 2019). The seeds usually germinate easily under proper storage conditions at 12°C and are able to retain their viability for up to 13 months (Kannan et al., 2013) and beyond, although improper or poor storage may lead to loss of viability in both species of barnyard millet. The seed dormancy, a major limiting factor in the cultivation of small millets, has not been studied yet in detail. However, in barnyard millet, both wild and freshly harvested seeds of cultivated species reported to have seed dormancy (Maun and Barrett, 1986; Sung et al., 1987; Manidool, 1992). Although the deep physiological dormancy in *E. crus-galli* grain was the most probable feature for its prolonged existence (Song et al., 2015), the innate dormancy present in cultivated *Echinochloa* species further hinders the evaluation or multiplication of seeds in germplasm conservation centers (Kovach et al., 2010). Despite this, the dormancy breaking treatments in *Echinochloa* also varies with species; some accessions may require light or dark and cold or heat or a combination of both (Kovach et al., 2010). Seed application of 100 ppm of IAA (Indoleacetic acid) improved germination percentage (18%), speed of germination (5.58 days earlier), and increased the seed length (11%), dry matter (3.80%), and vigor index (21%) (Sujatha et al., 2013). In another study, barnyard millet seeds treated with *Pseudomonas fluorescens* enhanced the seed germination and seedling growth attributes in barnyard millet (Sridevi and Manonmani, 2016). This is mainly due to the direct suppression of deleterious pathogens or the indirect production of growth hormones that ultimately increases the uptake, solubilization, and translocation of less available minerals (Olanrewaju et al., 2017).

*Echinochloa* species are generally considered to be a short-day plant (Muldoon, 1985) exhibiting photoperiodism and perform as per the different ranges of photoperiods from short days (8–13 h) to long days (16 h) (Maun and Barrett, 1986; Mitich, 1990). For instance, the variety CO (Kv) 2 reported having variable flowering times in temperate and hot regimes within regions of southern parts of India, with hindered uniform grain yield across the state (Vanniarajan et al., 2018). To alleviate that, the latest variety MDU 1, released in Tamil Nadu, India, has been found to have a short duration along with stable grain yield across the state (Vanniarajan et al., 2018).

## Relevance of Barnyard Millet in Climate Change and Nutritional Security

### Responses to Biotic and Abiotic Stresses

The *Echinochloa* species generally has potential resistance against various biotic and abiotic stresses. However, cultivated species such as *E. esculenta* and *E. frumentacea* are widely threatened by pest and diseases (i.e., shoot fly, stem borer, grain smut, and loose smut) at different growth stages of the crop (Jain et al., 1997; Jagadish et al., 2008). Aphid’s infection at the vegetative stage causes considerable yield reduction to *E. frumentacea*. So far, DHBM 996 and TNEF-204 were found to be resistant genotypes for shoot fly and stem borer (Rawat et al., 2019). Meanwhile, Kim et al. (2008) reported that some *E. frumentacea* accessions have the potential for antifeeding activity against brown plant hopper, which is among the major pests that affect rice production. On the other hand, loose smut (*Ustilago tritici*) and grain smut (*Ustilago panici frumentacea*) are major fungal diseases that affect the grain formation in both the cultivated species of *Echinochloa* (Jain et al., 1997; Gupta et al., 2010a). A heavy infestation of smuts during head formation leads to a significant reduction in grain yield and quality (Gupta et al., 2010a). However, Nagaraja and Mantur (2008) and Gupta et al. (2010a) showed that some of the *E. esculenta* accessions had the immunity against both smut diseases and further provide the chance to breed the resistance lines.

Abiotic stresses are a major threat to important food crops such as rice, wheat, and maize, and cause serious yield loss across the world. However, *Echinochloa* species have a high degree of tolerance to various abiotic stresses (Gupta et al., 2010b; Singh et al., 2010). For instance, a recent investigation from Arthi et al. (2019) showed that among the 89 *Echinochloa* accessions, CO (Kv) 2, MDU 1, PRJ1, TNEf 301, TNEf 204, TNEf 361, TNEf 364, and VL 29 exhibited better germination as compared to the rice variety, White Ponni, at 200 mM NaCl concentration. Similarly, *Echinochloa* species are also the preferable choice of farmers for cultivation in various adverse environments such as those prone to drought or flooding. These features showed that the *Echinochloa* species might have some specialized rhizosphere organizations that can facilitate the uptake and release of oxygen (O_2_) from their roots at stressful conditions. Zegada-Lizarazu and Iijima (2005) reported higher water uptake efficiency (deep root) of barnyard millet (*E. utilis*) over other minor millets, including pearl millet, and found that barnyard millet sustained and increased the water use efficiency, leaf area index, and dry matter production in both drought and flooding conditions. Therefore, it is also worth investigating the *Echinochloa* species mechanism behind the tolerance to drought and flooding stress. Further, identification and characterization of genes and pathways associated with resistance to saline, drought, and flooding stress in *Echinochloa* species may not only be useful to develop superior cultivars but also assist in improving the tolerance in a major cereal crop. It is also well known for its excellent nitrogen-use efficiency over cereal crops (Goron and Raizada, 2015) and has been recommended as a natural phyto-extractor in heavy metal (lead, cadmium, and chromium) contaminated soils and sodic soils due to hyper accumulation nature. Since heavy metals are currently of much environmental concern, phyto-based soil reclamation is an alternative, cost-effective, and eco-friendly approach (Subhashini and Swamy, 2014) that needs to be imparted in soil health restoration programs.

### Nutritional Significance and Health Benefits

In terms of nutritive value, barnyard millet is superior to major and minor millets. Barnyard millet grains are a rich source of dietary fiber, iron, zinc, calcium, protein, magnesium, fat, vitamins, and some essential amino acids (Singh et al., 2010; Saleh et al., 2013; Chandel et al., 2014). The nutritional composition of barnyard millet is presented in Supplementary Table S1. The average carbohydrate content of barnyard millet varies between 51.5 and 62.0 g/100 g (Saleh et al., 2013), which is lower than that of other major and minor millets. Ugare et al. (2014) reported that the crude fiber of barnyard millet is higher than any other cereal, ranging between 8.1 and 16.3%. The high ratio of carbohydrate to crude fiber ensures the slower release of sugars in the blood, and so thus aids in maintaining blood sugar level. The resistant starch in barnyard millet has shown to lower blood glucose, serum cholesterol, and triglycerides in rats (Kumari and Thayumanavan, 1998). In a clinical study with human volunteers, Ugare et al. (2014), confirmed a lower glycemic index (GI) in type 2 diabetic groups during regular consumption of barnyard millet meal. Existing evidence showed that the protein content (11.2–12.7%) in barnyard millet was reasonably higher than other major cereals and millets. Although the total minerals, ash, fat, and amino acid content in barnyard millet were although comparable with other cereals and millets, the iron content in the grain was significantly higher than others. For instance, the iron content in barnyard millet grain is about 15.6–18.6 mg/100 g (Saleh et al., 2013; Renganathan et al., 2017; Vanniarajan et al., 2018), which is rationally higher than major cereals and millets. In addition, a lower phytate (3.30–3.70 mg/100 g) content in grains (Panwar et al., 2016) followed by the dehulling process has also decreased phytic acids significantly, favoring the bioavailability of minerals. This makes barnyard millet an ideal food not only for people with lifestyle diseases, but also for anemic patients and especially women in developing countries. The polyphenols and carotenoids are known to have several potential benefits to humans, and are twofold higher in barnyard millet than finger millet (Panwar et al., 2016). Similarly, alkaloids, steroids, carbohydrates, glycosides, tannins, phenols, and flavonoids present in barnyard millet have various ethno-medical properties like being antioxidant, anti-carcinogenic, anti-inflammatory, antimicrobial, having a wound healing capacity, biliousness, and alleviating constipation-associated diseases (Kim et al., 2011; Ajaib et al., 2013; Moreno Amador et al., 2014; Borkar et al., 2016; Nguyen et al., 2016; Sharma et al., 2016; Sayani and Chatterjee, 2017). Collectively, all these features make the barnyard millet a suitable and secured food for present-day consumers in their overall physical and nutritional well-being.

### Physico-Chemical Properties and Relationship With Cooking and Value Addition

The studies on the physical and mechanical properties of grains are an important criterion to design any processing instruments like dehullers, polishers, sorters, storage, and other processing machineries (Singh et al., 2010). In barnyard millet, grain moisture is the prime criteria playing a key role not only in storage but also in the development of processing machineries. The moisture level of barnyard millet grain highly influences the quality as well as the time of milling and polishing (Lohani et al., 2012). For instance, the 8% moisture content of the grain is better for polishing than at 14% moisture. However, at 14% moisture, the degree of polishing increases grain recovery and decreases the loss of protein, fat, ash, and fiber. Based on this, Lohani et al. (2012) suggested 10% as the optimum moisture level for polishing. Similarly, physical (grain diameter, grain surface area, 1,000 grain mass, true density, dynamic angle of repose, coefficient of internal friction, and coefficient of static friction), aerodynamic (terminal velocity), and mechanical (specific deformation and rupture energy) properties are other major parameters influenced by the moisture content of the grains. All these factors ultimately influence the processing of grains in the machines (Singh et al., 2010). Therefore, a study in detail on these properties should be conducted in order to design and develop better milling, polishing, grading, and sorting machineries for barnyard millet.

The cooking and flour quality of the grain are primary standards to assess consumer acceptability. At the same time, different processing techniques aim to increase the storage time of grain/flour as well as increase the physicochemical accessibility of nutrients with reduced anti-nutrient losses during consumption. Barnyard millet grains are usually parboiled-dehulled-cooked and consumed in a similar way to rice (Surekha et al., 2013). It requires about 12 min to cook. The grain can also transform into flour for the preparation of various food formulation by processing techniques. Nevertheless, different processing methods cause the variations in the functional, nutritional, anti-nutritional, and pasting properties of barnyard millet flour (Nazni and Shobana, 2016). In addition, physical parameters, such as bulk density and porosity are important criteria in flour storage and oxidation related problems. Nazni and Shobana (2016) compared the flour of raw and germinated rice for setting up different processing methods for storage and transportation. The study found that the germinated flour exhibited decreased bulk density and porosity (air spacing) than raw rice flour. Therefore, the germinated flours are comparatively less prone to autoxidation than raw rice due to reduced air space between the flour molecules, and this could prevent the spoilage of flour and facilitate easy packaging and enable long-distance transportation. Apart from that, the germinated flours also have an increased oil absorption capacity that makes the flour suitable for enrichment in flavor and mouth feel. Similarly, value-added food products are not only free of anti-nutritional factors but also increase nutritional compounds, making barnyard millet a good base ingredient for infant food formulas. Besides, the flour is highly amenable for various food preparations such as baby foods, snacks, and dietary foods (Vijayakumar et al., 2009; Anju and Sarita, 2010; Surekha et al., 2013). The flour is also highly compatible to blend with other food flours for making novel or any value-added products without affecting the flavor and taste (Veena et al., 2004; Surekha et al., 2013). For instance, a ready-to-eat snack food can successfully be prepared with barnyard millet, potato mash, and tapioca powder in the ratio of 60:37:3 (Jaybhaye and Srivastav, 2015). However, tannin content (0.21–0.36%) in the grain affects the *in vitro* protein digestibility (IVPD). Although, compared to other small millets (kodo millet and finger millet), it was very low. Therefore, it is suggested that there are many chances for the application of several processing techniques to improve barnyard millet flour quality and nutritional properties, especially in the value-addition strategy of food industries.

### Versatile Research and Industrial Applications

The amylose-rich barnyard millet starch has now attracted attention in biodegradable film making industries as an antioxidant packaging material (Cao et al., 2017). The incorporation of borage seed oil in barnyard millet starch increases the elongation range and decreases the tensile strength, water permeability, and moisture content properties of the starch, which makes it suitable for biofilm production. These biofilms are found to be resistant against various microbes and block light transparency and free radical formation in food industries (Cao et al., 2017). Research on Nanoparticle by Kumar et al. (2016) suggested the use of an aqueous extract from aerial parts of *E. colona* plant in the synthesis of silver nanoparticles (AgNPs) as a new eco-friendly approach in bio-synthesizing nanoparticle in plants. Such synthesis of AgNPs from plant extracts could be a safe and eco-friendly approach with possibilities for application at large-scale in the near future in the field of medicine, engineering, and agriculture. Mosovska et al. (2010) reported that *E. esculenta* extract showed antimutagenicity against 3-(5-nitro-2-furyl) acrylic acid in strains of *Salmonella typhimurium* due to its higher polyphenolic content, thereby playing a major role as antioxidants in scavenging H_2_O_2_ radicals. Similarly, a novel antifungal peptide, *EcAMP1*, was identified in the seeds of *E. crus-galli*, a unique antimicrobial peptide with a wide spectrum of antifungal activity against phytopathogens such as *Alternaria*, *Botrytis*, *Fusarium*, and *Trichoderma.* This peptide has a unique disulfide stabilized α-helical hairpin structure that intensively binds to the surface of fungal conidia, accumulates in the cytoplasm, and finally inhibits the elongation of hyphae without lysis of the cytoplasmic membrane (Nolde et al., 2011). This property could be exploited in future protein engineering technologies for the synthesis of novel antimicrobials in the agriculture and pharmaceutical industries. Besides all these, Barnyard millet has a higher straw yield and fodder value even at multiple cuttings (Bandyopadhyay, 2009). The fodder yield is about 6.3 tons/ha (Vanniarajan et al., 2018). Fodder contains a good amount of protein (7.6%), digestible fiber (23%), ash (12%), and fat (2.0%). Besides its superior feed quality, higher digestibility and nitrogen concentrations have meant barnyard millet is used as a potential livestock feed crop in the dry areas of the Deccan plateau to the extreme hills of the temperate sub-Himalayan region (Singh and Singh, 2005; Bandyopadhyay, 2009; Yadav and Yadav, 2013; Sood et al., 2015).

## Germplasm Resources and Utilization

The major collections of barnyard millet germplasm accessions are housed in India and Japan. Vivekananda Parvatiya Krishi Anusandhan Sansthan (VPKAS), India; Indian Institute of Millets Research (IIMR), India; National Institute of Agrobiological Sciences (NIAS), Japan, and Consultative Group on International Agricultural Research like International Crop Research Institute for the Semi-Arid Tropics (ICRISAT), India are actively working on germplasm evaluation for various agronomic, biotic, and abiotic stresses, grain, and nutritional content traits in barnyard millet. India made a series of collaborative exploration missions (i.e., Indo-Australian missions, Indo-Japanese missions, and Indo-Soviet protocol) for the improvement of barnyard millet and other millets with different countries across the world. So far, six hundred and one exotic barnyard millet accessions have been introduced into India between the period of 1976 and 2007 to increase the food and fodder production (Gomashe, 2017). The major source of introduction was from Australia, Canada, France, Germany, Ghana, Italy, Japan, Kenya, Malawi, the Philippines, Russian Federation, South Africa, Spain, United States of America, and Yugoslavia. During this period, Indian barnyard millet accessions were also introduced in the United States, Canada, and Australia for feed and forage purposes (Gomashe, 2017). At present, 8,000 barnyard millet germplasms have been conserved at different centers throughout the world (Table 2).

**TABLE 2:** Major organizations across the globe conserving the *Echinochloa* species (till December 2019).

**Country**	**Number of accessions**	**Organization**
India	1888	National Bureau of Plant Genetic Resources, New Delhi
	985	University of Agricultural Sciences, Bangalore
	749	International Crop Research Institute for the Semi-Arid Tropics, Patancheru
	300	Vivekananda Parvatiya Krishi Anusandhan Sansthan, Almora
	1561	Indian Institute of Millets Research, Hyderabad
Japan	3671	National Institute of Agrobiological Sciences, Kannondai
	65	Plant Germplasm Institute, Kyoto University
United States	232	USDA Agricultural Research Service, Washington
	306	National Centre for Genetic Resources Conservation, Collins
	304	North Central Regional Plant Introduction Station, Ames
China	717	Institute of Crop Science, Chinese Academy of Agricultural Sciences, Beijing
Kenya	208	National Gene bank of Kenya, Crop Plant Genetic Resources Centre, Muguga
Ethiopia	92	International Livestock Research Institute, Addis Ababa
Australia	66	Australian Plant Genetic Resource Information Service, Queensland
Pakistan	50	Plant Genetic Resources Program, Islamabad
Norway	44	Svalbard Gene Bank, Spitsbergen
United Kingdom	44	Millennium Seed Bank Project, Seed Conservation Department, Royal Botanic Gardens, London
Germany	36	Leibniz Institute of Plant Genetics and Crop Plant Research, Gatersleben

**Source:*http://apps3.fao.org/wiews/germplasm_report.jsp?*

To obtain basic knowledge about germplasm, morphological characterization is the preliminary step for characterizing and classifying any collected/introduced materials. This not only provides the heritability of traits but also increases the utility of promising materials in breeding programs. In a study, Gupta et al. (2009) evaluated barnyard millet germplasm at the Himalayan regions of Uttarakhand, India, and identified some promising donors for plant height (<120 and >200 cm), productive tillers (>4), inflorescence length (>28 cm), raceme number (>50), raceme length (>3.1 cm), and grain yield (>16 g). In another study, to enable efficient use of genetic resources and to increase its access for breeders, barnyard millet core collection comprising of 89 accessions had also been established based on phenotypic and genotypic characterization (Upadhyaya et al., 2014). It was revealed that the mean difference between 89 core accessions and 736 whole accessions for most of the agronomic traits were not significant, indicating that the entire genetic variation had been sufficiently preserved in this core collection (Upadhyaya et al., 2014). The coefficient of variation among core germplasm varied from 0.79 to 36.43% for days to maturity and basal tiller number, while the heritability (broad sense) varied between 70.14 and 99.87% for inflorescence length and days to maturity, respectively.

Further, the multidimensional principal component analysis (PCA)-based phenotypic characterization of these 89 accessions resulted in three different groups for agronomic and other phenotypic traits based on its origin (Sood et al., 2015). The study has also identified some promising genotypes, which could be efficiently used in a breeding program for the improvement of early maturity, grain yield, and yield contributing traits. Similarly, the IIMR, Hyderabad, evaluated the 146 barnyard millet accessions and found a larger variation for grain yield and yield contributing traits, which led to the identification of 18 promising accessions for barnyard millet breeding programs (IIMR, 2016). A comparison of agronomic traits from various trials conducted across India is given in Supplementary Table S2, which revealed barnyard millet genotypes to have considerable variation for yield and yield-related traits. For instance, the genotypes with higher grain yield and yield contributing traits (panicle length, number of raceme, and grain yield) were identified in the Southern States of India viz., Telangana and Tamil Nadu. In contrast, early maturing (58–90 days) genotypes were mostly found in the Northern States of India.

On the other hand, registration of trait-specific germplasms in the National Gene Banks (NGB) not only protects the natural resources from Intellectual Property Rights (IPRs) but also facilitates the breeders to access important/valuable genotypes for any crop improvement programs. In crop plants, 60% of the registered traits of germplasms belong to cereals, oilseeds, and legumes for resistance against various biotic and abiotic stresses. With regard to cereals, the maximum number of germplasms were registered in paddy and wheat mainly for biotic stress related traits (Radhamani et al., 2011). However, in millets except for sorghum and pearl millet, most of them were registered with limited traits only, mainly, cytoplasmic male sterile (CMS) line in foxtail millet (Radhamani et al., 2011), waxy trait in proso millet (Santra et al., 2015), and easy de-hulling in barnyard millet. The registered barnyard millet genotype B29 by VPKAS, Almora, showed a 42–146.4% faster de-hulling percentage over other check varieties (Gupta et al., 2014). Therefore, despite the focus on higher grain yields alone, barnyard millet breeding programs should also include the strategy of registration of unique traits that might be conserved in the landraces, germplasms, or rejected entries from the evaluation trials.

The successful utilization of barnyard millet genetic resources resulted in the release of more than 20 varieties and cultures across India (Gomashe, 2017). The first variety of K1 was developed by the pureline selection method from local landraces of Tenkasi, Tamil Nadu, India, released during 1970, which possesses an average state yield of 1,000 kg ha^–1^. Later, several varieties were released against various pests and diseases across India through pureline selection from local landraces or exotic germplasm accessions. Among these, the notable variety PRJ 1, a direct selection from exotic collections of ICRISAT was released during 2003, by Vivekananda Institute of Hill Agriculture, Almora, Uttarakhand, India, possess a higher grain yield (2,500 kg ha^–1^) with resistance against various smuts (Upadhyaya et al., 2008). Recently, MDU 1, a variety developed by Agricultural College and Research Institute, Tamil Nadu Agricultural University, Madurai, India, through pureline selection of local landrace of Tamil Nadu possesses the characteristic features of short duration (<100 days) and higher grain yield (2,500 kg ha^–1^) (Vanniarajan et al., 2018). Besides, this variety also possesses a higher amount of iron content (16 mg/100 g) in the grains with good milling and cooking quality. In Japan, the “Noge-Hie,” a low amylose grain-containing cultivar was identified from a local landrace possessing natural deletion in one of three waxy genes (Hoshino et al., 2010). At the same time, “Chojuromochi,” a mutant developed through artificial γ-radiation, was completely devoid of Waxy (*Wx*) protein synthesis. In addition, the waxy protein trait was found to be stably inherited. Such glutinous variety in barnyard millet is in huge demand from Japanese consumers and industries for various food preparations similar to rice from paddy.

Hybridization is a difficult task in small millets, however, the hot water-based method followed by the contact method of crossing was found to be effective in finger millet (Raj et al., 1964; Nandini and Fakrudin, 1999) and foxtail millet (Siles et al., 2001). The same method was also applied in barnyard millet hybridization programs (Renganathan et al., 2015; Sood et al., 2015). Prior to pollination (early morning), the panicles which started flowering were selected for emasculation (Renganathan et al., 2015; Sood et al., 2015). The selected panicle was trimmed for 1/3rd portion by removing the opened and immature flowers in the respective upper and lower portions of the racemes, and then the remaining middle portion was immersed in hot water at 52°C in a thermos flask for 1 min. The emasculated panicles were then covered with butter paper bags to avoid contamination. The panicles in which flowering had already commenced were chosen as a pollinator source and panicle to panicle contacts were made by tying them together with a thread. The male and female panicles thus secured together were covered by a butter paper bag to avoid contamination with foreign pollen. However, priority should be given for the development of CMS line in barnyard millet for the better exploitation of variability as followed in foxtail millet.

## Genomic Resources and Utilization

Whole genome sequence (WGS) is fundamental to understand the genome composition and gene repertoire of a crop. It helps to identify important genes and pathways related to economically important traits in crops. Recent advances in second and third-generation sequencing technologies have facilitated simple and cost-effective sequencing platforms to generate genome and transcriptome sequences. Among millets, the whole genome sequencing was completed in sorghum, pearl millet, foxtail millet, finger millet, and proso millet by various researchers (Zhang et al., 2012; Mace et al., 2013; Hittalmani et al., 2017; Varshney et al., 2017; Zou et al., 2019). Genomic resources are also considerably well-defined in sorghum, pearl millet, foxtail millet, and finger millet due to the presence of genetic linkage maps, physical maps, cytogenetic stocks, and large-insert libraries (as reviewed by Varshney et al., 2006; Gomashe, 2017). However, in barnyard millet, very limited attempts have been made to discover the genomic structure and associated downstream processes due to its genome complexity and lack of research funding on this orphan crop.

### Chloroplast Genomes and Phylogeny Analysis

**FIGURE 5:**
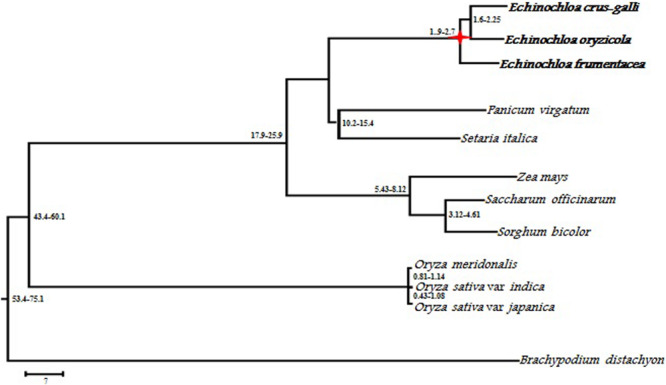
Phylogenetic tree showing relationships among *Echinochloa* species and grass species (from Perumal et al., 2016).

The chloroplast genome of *Echinochloa* is highly conserved for its genome structure, organization, and gene order (Ye et al., 2014). So far, chloroplast genomes of seven *Echinochloa* species including *E. crus-galli*, *E. ugandensis*, *E. stagnina*, *E. colona*, *E. esculenta*, *E. frumentacea*, and *E. oryzicola* (Ye et al., 2014; Perumal et al., 2016; Lee et al., 2017; Sebastin et al., 2019) have been sequenced. It revealed their genome structure (quadripartite), identity (99.5%), and size (139,592–139,851 bp) of the chloroplast genomes. The quadripartite structure generally confers a major impact on the evolution of plastome sequences of an organism (Yang et al., 2013). Such a quadripartite genome comprises a pair of inverted repeats (IR) separated by a small single-copy region (SSC) and a large-single copy region (LSC). The comparison of chloroplast genomes of wild and cultivated *Echinochloa* is given in Table 3. The size of IR, LSC, and SSC regions varied from 22,618 to 22,748, 81,837 to 82,053, and 12,518 to 12,519 bp, respectively. Similar to other angiosperms, the chloroplast genome of *Echinochloa* species comprises of 38.6% GC regions and 61% AT regions (Sebastin et al., 2019). In contrast, the number of genes varied from 111 to 131 among the species of *Echinochloa*, with the cultivated species exhibiting minimum. This could be mainly due to the reorganization of gene copy number and structure during the course of evolution or speciation. The divergence in copy number of any gene further creates the genetic polymorphism between the species, which contributes a major variation in their genome size and phenotype (Suryawanshi et al., 2016). As reported in angiosperms by Wendel (2015), the high morphological variation among the wild and its cultivated species occurs due to the consequences of genome reorganization during the evolutionary process. The comparative analysis of *Echinochloa* chloroplast genomes revealed that they are closer to the *Panicum virgatum* than other grasses (Ye et al., 2014). Further, molecular divergence clock analysis of grass species revealed that *Echinochloa* species had diverged 21.6 Mya than others. The wild species, such as *E. oryzicola* and *E. crus-galli*, diverged around 3.3 Mya while another wild species, *E. colona*, diverged from *E. oryzicola* and *E. crus-galli* between 2.65 and 3.18 Mya, respectively (Lee et al., 2017). Later, the cultivated species (*E. frumentacea*) diverged from *E. oryzicola* and *E. crus-galli* in and around 1.9–2.7 Mya (Perumal et al., 2016) (Figure 5). However, both wild and cultivated *Echinochloa* species have a high sequence identity with *P. virgatum* and *Sorghum bicolor* and low sequence identity with *Triticum aestivum* and *Oryza sativa* (Ye et al., 2014), which further concludes that *Echinochloa* species are more closely related to the *P. virgatum* and *S. bicolor* than *Triticum* and *Oryza.*

**TABLE 3:** Summary statistics of chloroplast genomes for wild and cultivated barnyard millet.

**Genome composition**	***E. crus-galli*^a^**	***E. colona*^b^**	***E. esculenta*^c^**	***E. frumentacea*^d^**
Genome size (bp)	139,851	139,592	139,851	139,593
Inverted Repeat size (bp)	22,640	22,618	22,748	22,618
Large-single copy region size (bp)	82,053	81,837	81,837	81,839
Small-single copy region size (bp)	12,518	12,519	12,518	12,518
Number of genes	131	131	111	112
Number of Protein coding genes	88	88	76	77
Number of tRNA	40	40	30	30
Number of rRNA	4	4	4	4
GC contents (%)	38.6	38.6	38.6	38.6

**^a^Ye et al., 2014; ^b^Lee et al., 2017; ^c^Sebastin et al., 2019; ^d^Perumal et al., 2016.**

### Transcriptome Analysis

With the advent of next-generation sequencing technologies (NGS), RNA-seq (RNA-sequencing) has now superseded the previous microarray technologies and a huge number of genomic resources are being generated in a cost and time effective manner (Weber, 2015). It not only generates differential genes, but also the functional molecular markers like simple sequence repeats (SSRs) and SNPs in various minor millet species. Enormous transcript profiles have been developed in weedy *Echinochloa* species till date for various traits associated with invasiveness and adaptations such as herbicide resistance, photosynthesis, flooding response, and other homeobox genes (Li et al., 2013a, b; Yang et al., 2013; Nah et al., 2015; Xu et al., 2015; Guo et al., 2017; Gao et al., 2018). Recently, transcriptome sequences developed from cultivated *E. frumentacea* variety CO (Kv) 2, yielded 97,065 transcripts with an average length of 94 Mbp (Murukarthick et al., 2019). Further *de novo* assembly, functional annotation, and comparison to *E. crus-galli* transcripts identified some key genes regulating Fe and Zn accumulation and drought tolerance. In addition, the study also generated 300 SSR primer pairs from 10,881 SSR loci targeting major repeats of trinucleotide (122) followed by dinucleotide (121), tetra-nucleotide (35), penta-nucleotide (20), and hexa-nucleotide (2).

A review of transcriptomes data in the NCBI database has revealed the presence of 952 gene sequences to date, generated from *E. crus-galli* (170), *E. oryzicola* (132), *E. frumentacea* (130), *E. esculenta* (130), *E. colona* (130), *E. ugandensis* (131), and *E. stagnina* (129). The details of transcriptome sequences published in the NCBI database were consolidated and presented in Table 4. Most of the genes were related to photosynthesis (PS I, PS II, NADH-plastoquinone *oxidoreductase*, ATP *synthase*), C4 pathways (phosphoenolpyruvate *carboxylase*, *aldolase*, *maturase* K, *kinase*), micronutrient transportation [Fe^2+^ transport protein 2-like protein (*IRT2*) gene, *nicotianamine synthase* 1 (*NAS1*), *nicotianamine synthase* 2 (*NAS2*), *polymerases* (RNA, DNA)], herbicide resistances (*1-aminocyclopropane-1-carboxylic acid synthase* 3, *acetolactate synthase*, calcineurin, cyclophilin 2, cytochrome P450, GH31, *glutathione S-transferase*), flooding tolerances (*enolase*, *alcohol dehydrogenase*), waxy grains (*granule-bound starch synthase*), non-shattering grains (*sh*_4_), ribosomal RNA, and transfer RNAs, etc. The proteomics exploration also revealed that a total of 540 proteins are found to be commonly expressed in *Echinochloa* species, of which most of the annotated protein sequences are tRNA, ribosomal, and other photosystem proteins. In addition, most of the proteins found in the *Echinochloa* species showed orthologs among themselves for proteins of C_4_ pathways, calcium binding protein, photosynthesis, *bZIP* transcription factor 1, translational initiation factors, transporters, and hypothetical proteins, etc. (Yang et al., 2013). However, some uniquely expressing proteins were also identified in the *Echinochloa* species. For instance, the maximum expression of quinclorac-resistant proteins, Cu/Zn superoxide *dismutase*, defensin, cadmium tolerant, viral nucleoprotein, and antimicrobial peptides was observed in *E. crus-galli* (Odintsova et al., 2008), multiple-herbicide-resistant proteins in *E. phyllopogan* (Iwakami et al., 2014), and *granule-bound starch synthase* in *E. esculenta* (Ishikawa et al., 2013). However, we require more research/data to draw a valid conclusion on the species-specific expression.

**TABLE 4:** Details of transcriptome sequences of *Echinochloa* species.

**Species**	**Parts**	**Data generated (Gb)**	**Platform**	**Accession number**
*E. frumentacea*	Leaf	18.3	ILLUMINA (Illumina HiSeq 2500)	SRX5210765
*E. frumentacea*	Leaf	48.6	ILLUMINA (NextSeq 500)	SRX3029505
*E. esculenta*	NA	11.6	ILLUMINA (Illumina HiSeq 2000)	SRX2698648
*E. crus-galli* var. *zelayensis*	Leaf	07.3	ILLUMINA (Illumina HiSeq 2000)	SRX3574154
*E. crus-galli*	NA	32.8	PacBio	SRX3081138
E. colona*	Leaf	11.6	ILLUMINA (Illumina HiSeq 2500)	SRX2588690
*E. stagnina*	Leaf	02.4	ILLUMINA (Illumina HiSeq 2500)	SRX3330321
*E. stagnina*	Roots	01.5	ILLUMINA (Illumina HiSeq 2500)	SRX3330365
*E. glabrescens*	NA	05.0	ILLUMINA (NextSeq 2000)	ERX990971
Barnyard millet core collection	NA	69.0	ILLUMINA (Illumina HiSeq 2500)	SRX734221

*^∗^Imazamox (herbicide) treated; NA, not available. Source: https://www.ncbi.nlm.nih.gov/search/all/?term=echinochloa..*

### Genome Sequence

Research conducted in China released the whole genome sequence of weedy *E. crus-galli* during 2017 and was annotated successfully for its unique nature of invasiveness and adaptation in the fields of crop plants (Guo et al., 2017). The total sequence length of the genome at a depth of 171× was estimated to the size of 1.27 Gb, representing around 90.7% of the predicted genome size. The genomic libraries range between^2^ 160 bp and 20 Kb with a total number of contigs of 4534 with minimum and maximum contigs size of 1 kb and 11.7 Mb, respectively. The gene annotation of *E. crus-galli* further revealed 108,771 protein-coding genes, 785 miRNAs, 514 Mb repetitive elements, and non-coding RNAs. As of 2019, the genomic resources available in the NCBI domain include 1,246 nucleotide sequences, 822 gene sequences, 2,468 protein sequences, 105 short read archive (SRA) sequences, 74 Expressed Sequence Tags (ESTs), and one Gene Expression Omnibus (GEO) dataset related to various species of *Echinochloa*. Among the species *E. crus-galli* (652), *E. oryzicola* (126), *E. phyllopogon* (96), *E. colona* (76), *E. pyramidalis* (46), *E. esculenta* (44), *E. frumentacea* (43), and *E. oryzoides* (32) hold the maximum number of sequences (Figure 3B). To date, 54% of nucleotide sequences are available for *E. crus-galli*, while cultivated barnyard millet *E. frumentacea* and *E. esculenta* have only 4%. The comparative scenario of genomic resources among small millets is presented in Supplementary Table S3, which further emphasized the need to enrich the cultivated barnyard millet genome in the future.

### Molecular Markers and Its Application

#### Genetic Diversity Analysis

Molecular markers are nucleotide sequences that are widely used for genetic diversity, linkage map construction, and marker assisted selection of crop plants (Muthamilarasan and Prasad, 2014). Early in the period of molecular marker research, Random Amplified Polymorphic DNA (RAPD) markers were utilized to access the genetic diversity and phylogeny among *Echinochloa* species (Hilu, 1994). Hilu (1994) proving that RAPD markers are effective in distinguishing both the cultivated and wild progenitors of the *Echinochloa* species at the genomic level. The genetic diversity among *E. frumentacea* was also found to be more diverse than *E. utilis* populations. However, isozyme marker analysis between these two species revealed that the accessions within the same species formed two different clusters and accessions from different species grouped into the same cluster, creating the possibility of the existence of intergrades and overlaps between the species (Prabha et al., 2010). Previously, Rutledge et al. (2000) obtained 90 polymorphic bands using 21 primer pairs with an average of 4.3 alleles per primer and Ruiz-Santaella et al. (2006) obtained 75 polymorphic bands using 13 primer pairs with an average of 5.8 alleles per primer. This suggests a low exhibition of polymorphism in the germplasm by the RAPD markers. The low level of polymorphism using RAPD markers has also been previously reported in finger millet diversity studies (Muza et al., 1995). Notwithstanding, the Amplified fragment length polymorphism (AFLP) marker system later developed a higher ability in revealing the genetic diversity in *Echinochloa* species compared to RAPD markers (Danquah et al., 2002; Tabacchi et al., 2009), since it generates more alleles per primer. For instance, a total of 166 polymorphic bands were produced in four primer pairs with an average of 41.5 per primer pairs in 28 genotypes. Whereas, the polymorphism information content (PIC) value of markers ranged between 0.44 and 0.52 (Kaya et al., 2014). This was in accordance with the previous report of Tabacchi et al. (2009), where seven primer pairs produced 156 polymorphic bands with an average of 22.3 alleles per primer in 80 genotypes. Similarly, polymerase chain reaction–restriction fragment length polymorphism (PCR-RFLP) techniques were successfully applied for species identification in *E. oryzicola* and *E. crus-galli* (Yamaguchi et al., 2005; Yasuda et al., 2006). Recently, InDel markers for *rbcL*, *matK*, and *ITS* genes identified in *E. colona* have been widely used as a cost effective approach in the DNA barcoding of *E. colona*, *E. oryzicola*, and *E. crus-galli* (Lee et al., 2014). All these studies emphasized that with insufficient sequence information, the RAPD and isozymes markers are the only choice, not only helpful in differentiating *Echinochloa* species, but also in laying the foundation for molecular breeding in barnyard millet.

Later on, a significant development in the sequencing technologies further eliminated the limitations present in the RAPD, RFLP, and AFLP techniques through sequence-based markers such as SSRs, EST-SSRs (Expressed sequence tags-simple sequence repeats), and SNPs. The sequenced-based markers are more desirable in genetic diversity studies due to their co-dominant, reproducible, highly polymorphic, and effective utilization in many crop plants (Lin et al., 2011). The information available on sequence-based markers in barnyard millet is still in its infancy, despite the reports of microsatellite markers related to genetic diversity studies in germplasm accessions gaining attention today. For instance, utilizing five SSR markers, 155 accessions of *Echinochloa* species including *E. esculenta* (49), *E. crus-galli* (94), and *E. esculenta* var. *formosensis* (12) were grouped into three separate clusters (Nozawa et al., 2006). The same study reported that the accessions belonging to *E. esculenta* were less diverse than those of *E. crus-galli* or *E. esculenta* var. *formosensis*. More recently, the ESTs markers also been proven to be a very informative and effective tool for the analysis of genetic diversity in many small millets. Extensive transcriptomics and annotation studies previously performed on herbicide resistant varieties of *E. crus-galli* resulted in 74 ESTs (Li et al., 2013b; Yang et al., 2013). However, those ESTs were limitedly used in the marker development and diversity studies in barnyard millet, since they are weedy ancestors. For instance, the *in silico* mining of *E. crus-galli* ESTs resulted in the identification of 22 pairs of EST-SSR primers (Babu and Chauhan, 2017). The study also reported that frequent SSR repeats were found to be tetra-nucleotide repeat followed by the penta- and hexa- nucleotide repeats. Among the repeats, GT (dimer), AGG and AGA (trimer), CAAA (tetra), TGTTT (penta), and AGACGA (hexa) were the most common type of repeat motifs in barnyard millet. On the other hand, a restriction-site associated DNA (RAD) approach combined with Illumina DNA sequencing strategy was performed in *E. phyllopogon* for the rapid and mass discovery of SSR and SNP markers by Chen et al. (2017). This study yields sequencing reads of 4132 contigs, of which 4710 are annotated to be putative SSRs and 49,179 are probable SNPs. Out of 4710 putative SSR markers, 78 were potentially polymorphic. Besides, the most frequent motif was AT and maximum motif length was dinucleotide type (>82%) followed by tri, tetra, penta, and hexa. The further validation of eight SSRs in four *E. phyllopogon* population resulted in 66 alleles with an average of 3.1–4.8 alleles from locus per population. Moreover, the study also identified a higher percentage of GC (48.9%) content in the genome, proving their successful nature of adaptation against freezing and desiccation, with GC% indicating more stability in an organism. Hence, SSRs and SNPs markers developed from *E. phyllopogon* may be very useful in studying not only the diversity, origin, and distribution of herbicides-resistant population (Osuna et al., 2011; Okada et al., 2013), but also for predicting gene location and molecular breeding in cultivated types. Recently, Manimekalai et al. (2018) and Murukarthick et al. (2019) used EST-SSR markers developed from the cultivated, *E. frumentacea* transcriptome sequence to analyze the genetic diversity of Indian barnyard millet germplasm. Manimekalai et al. (2018) used 51 EST-SSR markers to study the genetic diversity of 61 barnyard millet germplasms. Among 51 EST-SSR markers, 14 were polymorphic and produced 29 alleles with the PIC value ranging between 0.276 and 0.652. Similarly, Murukarthick et al. (2019) identified 10 polymorphic markers from 30 EST-SSRs and showed clear polymorphism in the 30 Indian barnyard millet germplasms.

Apart from SSR markers, a total of 21,000 SNPs were identified and characterized through the whole-genome genotyping-by-sequencing (GBS) method using core germplasm comprising of 95 barnyard millet accession (Wallace et al., 2015). About 10,816 out of 21,000 SNPs were spread across 65 biotypes of *E. colona*, and 8,217 SNPs across 22 biotypes of *E. crus-galli*. The SNPs discriminating among *E. colona* and *E. crus-galli* biotypes were 1,299 and 1,444, respectively. Further, population structure analysis with SNPs strongly separated these two species with four clusters in *E. colona* and three clusters in *E. crus-galli* (Wallace et al., 2015).

#### Gene/QTL Mapping

The use of molecular markers such as SSRs and SNPs provide opportunities for breeders to identify the QTL/gene(s) for important micronutrient and agronomical traits in barnyard millet. So far, many SSR and SNP markers (Wallace et al., 2015; Chen et al., 2017; Manimekalai et al., 2018; Murukarthick et al., 2019) have been developed to speed up the linkage map construction and QTL mapping in barnyard millet, but no genetic linkage map or QTLs published yet compares to other millets such as foxtail millet and finger millet (Supplementary Table S4). To date, two mapping studies only have been published on barnyard millet (Table 5). Ishikawa et al. (2013) identified functional SNP markers for waxy traits and found that these waxy traits are controlled by three loci, namely *EeWx1*, *EeWx1*, and *EeWx3*. The plants with functional alleles in all three loci exhibited normal amylose content (wild), while any one of the alleles (natural mutant) and/or completely homozygous mutant alleles (artificial mutant) exhibited low amylose and very low amylose content (waxy), respectively. Besides, the phylogenetic analysis also revealed that the waxy gene sequences are highly conserved among grass species. In another study, bulk segregant analysis (BSA) and 51 EST-SSR markers were used to analyze the F_2_ individuals of ACM 331 × MA 10, contrast parents for anthocyanin pigments, and the results showed that the SSR marker, BMESSR 39, was linked with anthocyanin pigments in barnyard millet (Renganathan et al., 2019). Conclusively, progress in barnyard millet genome mapping remains slow and is still in its initial stage. Moreover, these published two reports contain preliminary results only; further experimental investigation is required to apply for marker-assisted selection (MAS).

**TABLE 5:** Molecular markers associated with waxy and anthocyanin pigment traits in barnyard millet.

**Markers**	**Trait**	**F_2_ Mapping population**	**Segregation ratio (χ^2^ analysis)**	**References**
SNP	Waxy	Wild × Mutant (γ) and Natural mutant × Mutant (γ)	3:1 and 63:1	Ishikawa et al., 2013
ESTSSR	Anthocyanin pigment	Pigmented × Green	1:2:1	Renganathan et al., 2019

**SNP, single nucleotide polymorphism; ESTSSR, EST-derived simple sequence repeat, EMS, ethyl methane sulfonate.**

### Comparative Genomics and Synteny Analyses

Comparative genomics studies brought considerable benefit to barnyard millet crop. Mainly, SSR markers obtained from the cereals and millets were successfully utilized to characterize the barnyard millet germplasm. The summary of cross transferable molecular markers developed in other cereals and millet are presented in Table 6. Due to the non-availability of whole genome sequencing in barnyard millet, the genomes of rice, maize, finger millet, and foxtail millet have served as essential models to study the marker-based syntenic relationships. The genomic SSR (gSSR) markers developed through *in silico* mining of the foxtail millet sequence showed a high degree of cross-transferability in barnyard millet and other related small millet species. Among the 159 gSSRs, 58 were found to show consistent amplification in barnyard millet, that is, 91.3% cross-species amplification ability (Pandey et al., 2013). Similarly, 106 eSSR (EST-derived simple sequence repeats) markers from *Setaria* showed consistent amplification in millet and non-millet species and also exhibited high cross species transferability in barnyard millet (90.6%) (Kumari et al., 2013). Muthamilarasan and Prasad (2014) reported that 100 out of 5,000 intron-length polymorphic markers (ILP) mined from the foxtail millet genome showed amplification in various small millets with 94 percentage cross-transferability in barnyard millet. Yadav et al. (2014), also found that the rice genic SSR primers from calcium transporters and calcium kinases group showed 100% and 72.2% cross transferability, respectively, in barnyard millet.

**TABLE 6:** Transferability details of cross cereal species markers to barnyard millet.

**Species**	**Marker**	**Number of amplified markers**	**PIC**	**Polymorphic marker**	**Cross-transferability (%)**	**References**
Finger millet	eSSR	15/104	NA	NA	14.2	Arya et al., 2014
	gSSR	20/132	NA	NA	15.5	Arya et al., 2014
	eSSR, gSSR	33/101	NA	NA	55.4	Krishna et al., 2018
	SSR	07/18	0.16–0.53	06	85.7	Babu et al., 2018a
	SSR	32/32	0.27–0.73	29	90.6	Babu et al., 2018b
Pearl millet	SSR	10/32	NA	NA	31.2	Arya et al., 2014
Foxtail	SSR	53/58	NA	NA	91.3	Pandey et al., 2013
	eSSR	106	0.00–0.48	NA	90.6	Kumari et al., 2013
	ILP	94/100	0.03–0.47	NA	94.1	Muthamilarasan and Prasad, 2014
	SSR	46/64	0.02–0.66	NA	73.4	Gupta et al., 2013
	SSR	7/26	NA	2	65.38	Krishna et al., 2018
Sorghum	eSSR	42	NA	NA	80.9	Yadav et al., 2014
Rice	eSSR	102	NA	NA	72.1	Yadav et al., 2014
	SSR	85/120	0.15–0.67	41	48.2	Babu et al., 2018b
Maize	SSR	32/46	0.25–0.73	26	70.0	Babu et al., 2018a

**SSR, simple sequence repeat; eSSR, EST-derived simple sequence repeat; gSSR, genomic simple sequence repeat; ILP, intron length polymorphism; PIC, polymorphic information content; NA, not available.**

The orthologs and paralogs analyses of the genome *E. crus-galli* against some of the grass family revealed that the approximate divergence times of *Oryza* – *Sorghum* and *Sorghum – Echinochloa* were estimated to be 48.5 and 28.5 Mya, respectively, followed by the polyploidization and speciation events by 7.8 Mya (Guo et al., 2017). Three copies of gene clusters related to the biosynthesis of DIMBOA (2,4-dihydroxy-7-methoxy-1,4-benzoxazin-3-one), a unique allelopathic compound reported in maize (Frey et al., 2009), were found in the *E. crus-galli* genome and each of them showed a perfect synteny with segments of BX1-5 and BX8 of the maize genome. Similarly, *E. crus-galli* also exhibited synteny with rice for momilactones, a phytoalexin compound expressed to protect against blast pathogens (Guo et al., 2017). Babu and Chauhan (2017) also found homology of some barnyard millet ESTs against the chromosomal regions of 2, 5, 6, 8, 9, and 12 of rice, the waxy gene of maize, *granule-bound starch synthase* I (GBSSI-S) gene of *Panicum repens*, *Setaria italica*, *Panicum miliaceum*, and super oxide *dismutase* (SOD) gene of *Colletotrichum eremochloae*. On the other hand, Babu et al. (2018a;b) compared rice, maize, and finger millet gSSRs for cross species amplification in barnyard millet and reported that maize and finger millet SSRs exhibited higher PIC values, efficient cross species amplification, and polymorphism percentage than rice SSRs. However, the comparative genetic mapping between rice and barnyard millet showed several putative syntenic regions across the genome that regulated the traits including seed dormancy, plant height, panicle length, spikelet characters, leaf senescence, seed weight/yield-related traits, shattering character, root traits, blast resistance, brown plant hopper (BPH) resistance, and amylose content (Babu et al., 2018b). Eventually, using the available literature in the published reports, we concluded that EST-derived SSR markers had higher cross-genome amplification than genomic SSR markers, indicating higher conservation of the former than the latter across the species of the grass family. Therefore, cross transferability mechanisms could be exploited in barnyard millet for trait-based marker identification.

## Conclusion and Future Prospects

Despite its nutritional and agronomic benefits, barnyard millet has remained an underutilized crop and has received very little attention from researchers as well as farmers across the globe. Barnyard millet breeding programs have stagnated due to limited funding from various funding agencies and research organizations. Therefore, considerable efforts are needed to develop varieties or hybrids with farmer/consumer preferred traits. More breeding programs have to be designed in the future for harnessing the genetic variability for high yield potential, yield stability, improved salinity tolerance, pest and disease resistance, as well as enhanced nutritional quality, especially micronutrient composition. However, the progress of barnyard millet breeding programs is very slow due to the lack of genetic and genomic resources. With respect to genetic resources, the size of the core collection in barnyard millet is comparatively less than that of other minor millets (foxtail and finger millet) and so far, breeding populations have not been developed. Therefore, core and mini-core collections representing maximum diversity as well as bi-parental and multiparent populations have to be established and evaluated for various nutritional and agronomic traits. These resources will be useful to track the genomic regions associated with targeted traits by the linkage-based QTL mapping, genome-wide association study (GWAS), and genomic selection (GS), as well as for the detection of candidate genes.

Despite genome research in barnyard millet being at its infancy and far behind other minor millets, transcriptome sequencing has allowed researchers to develop several genomic resources, including EST-SSRs and SNPs, that could be useful for marker-assisted breeding. However, extensive efforts are needed in the future to develop the reference genome, genome-wide SSR and SNP markers, construction of genetic linkage maps, and physical maps. The recent release of the genome sequence of a weedy ancestor (*E. crus-galli*), together with the genomic resources from major and minor millet crops, offers an initial framework for enriching genomic research in an orphan crop like barnyard millet by comparative genomic approaches. It is also fruitful to use the *E. crus-galli* genome as a reference genome for cultivated barnyard millet species similar to the case in bread wheat. It helps not only to understand the genome composition of cultivated barnyard millet species and increases mapping accuracy, but also helps us to know the effect of variants on protein function.

**FIGURE 6:**
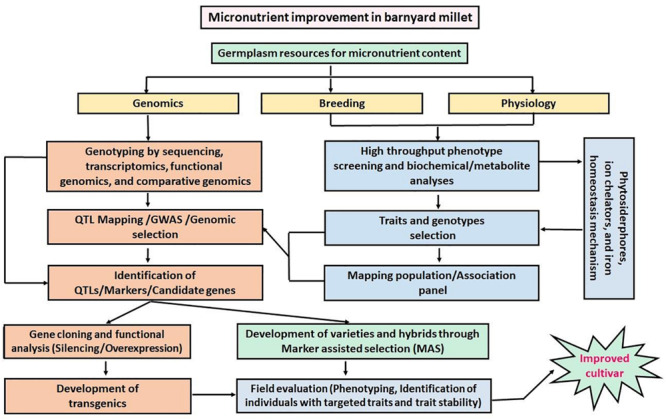
Schematic representation of the proposed strategy for micronutrient improvements in barnyard millet.

Barnyard millet is a potential crop for the biofortification of micronutrients. The grains of barnyard millet are rich in micronutrients (Fe and Zn) and hence, the identification of potential genes related to the accumulation of micronutrients (Fe and Zn) will be helpful to transfer these genes to high yielding barnyard millet cultivars or even to other major crops like rice, wheat, maize, etc. Strategies for the improvement of micronutrients in barnyard millet are presented in Figure 6, which are also applicable to other agronomic traits. Furthermore, barnyard millet is well adapted to both warm and temperate regions and it is a rich source of genes responsible for stress tolerance. Therefore, understanding the molecular mechanism of plant responses to stress in inherently stress-tolerant crops such as barnyard millet will be useful in developing highly stress-tolerant cultivars. So far, several stress tolerance genes were identified in barnyard millet, but the function of these genes has not been tested by overexpression studies, mainly due to the lack of a genetic transformation system. To date, very limited reports have been published on genetic transformation in barnyard millet. Therefore, there is an immense need to develop an efficient transformation system for barnyard millet in the future so that it also paves the way for functional genomics studies related to tolerance against biotic and abiotic stresses as well as micronutrient traits.

Besides these research gaps, the farming community is still unaware of the true potential of barnyard millet cultivation in terms of nutritional value and productivity. Farmers generally cultivate this crop under marginal areas, but they still depend on low yielding local landraces. Therefore, support from non-government organizations (NGOs) can help in increasing awareness among the farmers, stakeholders, nutritionists, and consumers to adopt and promote barnyard millet cultivation as well as consumption. Moreover, being a polyploid, ratooning (or) multi-cutting practices have to be standardized, like in sorghum, for better utilization of the growing season for grain and green fodder production. There is also an urgent need for advancements in post-harvest technologies for better processing and value-addition in the barnyard and other minor millets. At the same time, a change in consumer preference toward small millets with simultaneous development of suitable food products, along with an increase in market price, would fetch better returns for farmers and healthier choices for consumers. Finally, when these challenges are overcome, barnyard millet, being nutritionally sound and environmentally hardy, is going to be a promising crop for sustainable food and nutritional security in future climate scenarios.

## Author Contributions

VR built the layout of the manuscript, collected literature, and wrote the manuscript. CV, JR, and AK provided suggestions. VR and AK revised and prepared the final draft of the manuscript.

## Conflict of Interest

The authors declare that the research was conducted in the absence of any commercial or financial relationships that could be construed as a potential conflict of interest.

## Footnotes

1 http://www.ncbi.nlm.nih.gov/sites/entrez

2 https://www.ncbi.nlm.nih.gov/sra?

## References

[B1] Ajaib M. Khan K. M. Perveen S. Shah S. (2013). Antimicrobial and antioxidant activities of *Echinochloa colona* (Linn.) link and *Sporobolus coromandelianus* (Retz.) kunth. *J. Chem. Soc. Pakistan.* 35 1384–1398.

[B2] Altop K. E. Mennan H. (2011). Genetic and morphologic diversity of *Echinochloa crus*-galli populations from different origins. *Phytoparasitica* 39 93–102. 10.1007/s12600-010-0135-3

[B3] Anju T. Sarita S. (2010). Suitability of foxtail millet (*Setaria italica*) and barnyard millet (*Echinochloa frumentacea*) for development of low glycemic index biscuits. *Malays. J. Nutr.* 16 361–368. 22691989

[B4] Aoki D. Yamaguchi H. (2008). Genetic relationship between Echinochloa crus-galli and Echinochloa oryzicola accessions inferred from internal transcribed spacer and chloroplast DNA sequences. *Weed Biol. Manag.* 8 233–242. 10.1111/j.1445-6664.2008.00303.x

[B5] Arthi N. Rajagopal B. Geethanjali S. Nirmalakumari A. Natesan S. (2019). Screening of barnyard millet (*Echinochloa frumentacea*) germplasm for salinity tolerance. *Electron. J. Plant Breed.* 10 659–666. 10.5958/0975-928X.2019.00083.8

[B6] Arya L. Chauhan D. Yadav Y. Verma M. (2014). “Transferability of simple sequence repeat (SSR) markers developed in Finger Millet, and Pearl Millet to Kodo Millet and barnyard Millet,” in *Innovative Approach in Stem Cell Research, Cancer Biology and Applied Biotechnology*, eds Johri A. K. Mishra G. C., (New Delhi: Excellent Publishing House)60–64.

[B7] Babu B. K. Agrawal P. K. Pandey D. Jaiswal J. P. Kumar A. (2014a). Association mapping of agro-morphological characters among the global collection of finger millet genotypes using genomic SSR markers. *Mol. Biol. Rep.* 41 5287–5297. 10.1007/s11033-014-3400-6 24861452

[B8] Babu B. K. Agrawal P. Pandey D. Kumar A. (2014b). Comparative genomics and association mapping approaches for opaque2 modifier genes in finger millet accessions using genic, genomic and candidate gene-based simple sequence repeat markers. *Mol. Breed.* 34 1261–1279. 10.1007/s11032-014-0115-2

[B9] Babu B. K. Dinesh P. Agrawal P. K. Sood S. Chandrashekara C. Bhatt J. C. (2014c). Comparative genomics and association mapping approaches for blast resistant genes in finger millet using SSRs. *PLoS One* 9:e99182. 10.1371/journal.pone.0099182 24915067 PMC4051690

[B10] Babu B. K. Pandey D. Agrawal P. K. Sood S. Kumar A. (2014d). In-silico mining, type and frequency analysis of genic microsatellites of finger millet (Eleusine coracana (L.) Gaertn.): a comparative genomic analysis of NBS–LRR regions of finger millet with rice. *Mol. Biol. Rep.* 41 3081–3090. 10.1007/s11033-014-3168-8 24477586

[B11] Babu B. Chauhan R. (2017). In-Silico Identification of EST Based Microsatellite Markers and SNPs, and Comparative Genomic Analysis of ESTs in Barnyard Millet for their Omics Applications. *Curr. Agric. Res. J.* 5 279–287. 10.12944/CARJ.5.3.03

[B12] Babu B. Rashmi C. Sood S. (2018a). Cross transferability of finger millet and maize genomic SSR markers for genetic diversity and population structure analysis of barnyard millet. *Indian J. Genet. Plant Breed.* 78 364–372. 10.31742/IJGPB.78.3.5

[B13] Babu B. Sood S. Kumar D. Joshi A. Pattanayak A. Kant L. (2018b). Cross-genera transferability of rice and finger millet genomic SSRs to barnyard millet (*Echinochloa* spp.). *3 Biotech* 8:95. 10.1007/s13205-018-1118-1 29430357 PMC5796944

[B14] Babu B. K. Joshi A. Sood S. Agrawal P. K. (2017). Identification of microsatellite markers for finger millet genomics application through cross transferability of rice genomic SSR markers. *Indian J. Genet.* 77 92–98.

[B15] Bai H. Cao Y. Quan J. Dong L. Li Z. Zhu Y. (2013). Identifying the genome-wide sequence variations and developing new molecular markers for genetics research by re-sequencing a landrace cultivar of foxtail millet. *PLoS One* 8:e73514. 10.1371/journal.pone.0073514 24039970 PMC3769310

[B16] Bajwa A. Jabran K. Shahid M. Ali H. H. Chauhan B. Ehsanullah (2015). Eco-biology and management of *Echinochloa crus*-galli. *Crop Prot.* 75 151–162. 10.1016/j.cropro.2015.06.001

[B17] Bandyopadhyay B. B. (1999). Genotypic differences in relation to climatic adaptation of two cultivated barnyard millet at Garhwal hills. *Indian J. Genet.* 59 105–108.

[B18] Bandyopadhyay B. B. (2009). Evaluation of barnyard millet cultivars for fodder yield under single and double cut treatments at higher elevation of hills. *Agric. Sci. Dig.* 29 66–68.

[B19] Barrett S. H. (1983). Crop mimicry in weeds. *Econ. Bot.* 37 255–282. 10.1007/bf02858881

[B20] Bennetzen J. L. Schmutz J. Wang H. Percifield R. Hawkins J. Pontaroli A. C. (2012). Reference genome sequence of the model plant Setaria. *Nat. Biotechnol.* 30 555–561. 10.1038/nbt.2196 22580951

[B21] Borkar V. S. Senthil Kumaran K. Senthil Kumar K. L. Gangurde H. H. Chordiya M. A. (2016). Ethno medical properties of *Echinochloa colona* and *Hydrolea zeylanica*: a review. *World J. Pharmaceut. Res.* 5 354–360.

[B22] Cao T. L. Yang S. Y. Song K. B. (2017). Characterization of barnyard millet starch films containing borage seed oil. *Coatings* 7:183 10.3390/coatings7110183

[B23] Chandel G. Meena R. Dubey M. Kumar M. (2014). Nutritional properties of minor millets: neglected cereals with potentials to combat malnutrition. *Curr. Sci.* 107 1109–1111.

[B24] Chauhan B. Johnson D. E. (2011). Ecological studies on Echinochloa crus-galli and the implications for weed management in direct-seeded rice. *Crop Prot.* 30 1385–1391. 10.1016/j.cropro.2011.07.013

[B25] Chen G. Zhang W. Fang J. Dong L. (2017). Identification of massive molecular markers in *Echinochloa phyllopogon* using a restriction-site associated DNA approach. *Plant Divers.* 39 287–293. 10.1016/j.pld.2017.08.004 30159521 PMC6112297

[B26] Cho Y. I. Chung J. W. Lee G. A. Ma K. H. Dixit A. Gwag J. G. (2010). Development and characterization of twenty-five new polymorphic microsatellite markers in proso millet (*Panicum miliaceum* L.). *Genes Genomics* 32 267–273. 10.1007/s13258-010-0007-8

[B27] Cidade F. W. de Souza-Chies T. T. Batista L. A. R. Dall’agnol M. Zucchi M. I. Jungmann L. (2009). Isolation and characterization of microsatellite loci in Paspalum notatum Flüggé (Poaceae). *Conserv. Genet.* 10 1977–1980. 10.1007/s10592-009-9872-6

[B28] Cidade F. W. de Souza-Chies T. T. Souza F. H. D. Batista L. A. R. Agnol M. D. Valls J. F. M. (2010). Microsatellite loci for Paspalum atratum (Poaceae) and cross-amplification in other species. *Am. J. Bot.* 97 107–110. 10.3732/ajb.1000207 21616809

[B29] Cidade F. W. Vigna B. B. Z. de Souza F. H. D. Valls J. F. M. Dall’Agnol M. Zucchi M. I. (2013). Genetic variation in polyploid forage grass: assessing the molecular genetic variability in the *Paspalum* genus. *BMC Genet.* 14:50. 10.1186/1471-2156-14-50 23759066 PMC3682885

[B30] Clayton W. D. Renvoize S. A. (2006). *Genera Graminum: Grasses of the world in Kew Bulletin Additional Series XIII, Royal Botanical Gardens Kew.* Chicago, IL: University of Chicago Press.

[B31] Danquah E. Y. Hanley S. J. Brookes R. C. Aldam C. Karp A. (2002). Isolation and characterization of microsatellites in *Echinochloa* (L.) Beauv. spp. *Mol. Ecol. Notes* 2 54–56. 10.1046/j.1471-8286.2002.00144.x

[B32] De Wet J. Prasada Rao K. Mengesha M. Brink D. (1983). Domestication of mawa millet (*Echinochloa colona*). *Econ. Bot.* 37 283–291. 10.1007/BF02858883

[B33] Denton D. C. (1987). “Food crops for waterfowl,” in *Fireside Waterfowler: Fundamentals of Duck and Goose Ecology*, eds Wesley D. E. Leitch W. G., (Mechanicsburg, PA: Stackpole Books), 352.

[B34] Dida M. M. Srinivasachary, Ramakrishnan S. Bennetzen J. L. Gale M. D. Devos K. M. (2007). The genetic map of finger millet, *Eleusine coracana*. *Theor. Appl. Genet.* 114 321–332. 10.1007/s00122-006-0435-7 17103137

[B35] Doggett H. (1989). “Small millets-a selective overview,” in *Small Millets in Global Agriculture*, eds Seetharam A. Riley K. W. Harinarayana G., (Oxford: Oxford), 3–18.

[B36] Fang X. Dong K. Wang X. Liu T. He J. Ren R. (2016). A high density genetic map and QTL for agronomic and yield traits in Foxtail millet [*Setaria italica* (L.) P. Beauv.]. *BMC Genomics* 17:336. 10.1186/s12864-016-2628-z 27146360 PMC4857278

[B37] Frey M. Schullehner K. Dick R. Fiesselmann A. Gierl A. (2009). Benzoxazinoid biosynthesis, a model for evolution of secondary metabolic pathways in plants. *Phytochemistry* 70 1645–1651. 10.1016/j.phytochem.2009.05.012 19577780

[B38] Gao Y. Li J. Pan X. Liu D. Napier R. Dong L. (2018). Quinclorac resistance induced by the suppression of the expression of 1-aminocyclopropane-1-carboxylic acid (ACC) synthase and ACC oxidase genes in *Echinochloa crus-galli* var. zelayensis. *Pestic. Biochem. Physiol.* 146 25–32. 10.1016/j.pestbp.2018.02.005 29626989

[B39] Gimode D. Odeny D. A. de Villiers E. P. Wanyonyi S. Dida M. M. Mneney E. E. (2016). Identification of SNP and SSR markers in finger millet using next generation sequencing technologies. *PLoS One* 11:e0159437. 10.1371/journal.pone.0159437 27454301 PMC4959724

[B40] Gomashe S. S. (2017). Barnyard millet: present status and future thrust areas. *Millets Sorghum Biol. Genet. Improv.* 134 184–198. 10.1002/9781119130765.ch7

[B41] Goron T. L. Raizada M. N. (2015). Genetic diversity and genomic resources available for the small millet crops to accelerate a New Green Revolution. *Front. Plant Sci.* 6:157. 10.3389/fpls.2015.00157 25852710 PMC4371761

[B42] Guo L. Qiu J. Ye C.-Y. Jin G. Lingfeng M. Zhang H. (2017). Echinochloa crus-galli genome analysis provides insight into its adaptation and invasiveness as a weed. *Nat. Commun.* 8:1031. 10.1038/s41467-017-01067-5 29044108 PMC5647321

[B43] Gupta A. Joshi D. Mahajan V. Gupta H. (2010a). Screening barnyard millet germplasm against grain smut (Ustilago panici-frumentacei Brefeld). *Plant Genet. Resour.* 8 52–54. 10.1017/S1479262109990141

[B44] Gupta A. Mahajan V. Gupta H. S. (2010b). “Genetic resources and varietal improvement of small millets for Indian Himalaya,” in *Biodiversity Potentials of the Himalaya*, eds Tewari L. M. Pangtey Y. P. S. Tewari G., (Gorakhpur: Gyanodaya Prakashan press), 305–316.

[B45] Gupta A. Mahajan V. Kumar M. Gupta H. (2009). Biodiversity in the barnyard millet (*Echinochloa frumentacea* Link. Poaceae) germplasm in India. *Genet. Resour. Crop Evol.* 56 883–889. 10.1007/s10722-009-9462-y

[B46] Gupta A. Sood S. Agrawal P. K. Bhatt J. C. (2014). B 29: an Easy Dehulling Barnyard Millet (*Echinochloa frumentacea* Link) Genotype. *Natl. Acad. Sci. Lett.* 38 21–24. 10.1007/s40009-014-0295-7

[B47] Gupta P. Raghuvanshi S. Tyagi A. (2001). Assessment of the efficiency of various gene promoters via biolistics in leaf and regenerating seed callus of millets, eleusine coracana and *Echinochloa crusgalli*. *Plant Biotechnol.* 18 275–282. 10.5511/plantbiotechnology.18.275

[B48] Gupta S. Kumari K. Muthamilarasan M. Alagesan S. Prasad M. (2013). Development and utilization of novel SSRs in foxtail millet [*Setaria italica* (L.) P. Beauv.]. *Plant Breed.* 132 367–374. 10.1111/pbr.12070

[B49] Hilu K. (1994). Evidence from RAPD markers in the evolution of *Echinochloa millets* (Poaceae). *Plant Syst. Evol.* 189 247–257. 10.1007/BF00939730

[B50] Hittalmani S. Mahesh H. B. Shirke M. D. Biradar H. Uday G. Aruna Y. R. (2017). Genome and Transcriptome sequence of Finger millet (*Eleusine coracana* (L.) Gaertn.) provides insights into drought tolerance and nutraceutical properties. *BMC Genomics* 18:465. 10.1186/s12864-017-3850-z 28619070 PMC5472924

[B51] Hoshino T. Nakamura T. Seimiya Y. Kamada T. Ishikawa G. Ogasawara A. (2010). Production of a fully waxy line and analysis of waxy genes in the allohexaploid crop, Japanese barnyard millet. *Plant Breed.* 129 349–355.

[B52] Hou S. Sun Z. Li Y. Wang Y. Ling H. Xing G. (2017). Transcriptomic analysis, genic ssr development, and genetic diversity of proso millet (*Panicum miliaceum*;Poaceae). *Appl. Plant Sci.* 5:1600137. 10.3732/apps.1600137 28791202 PMC5546162

[B53] Hunt H. V. Denyer K. Packman L. C. Jones M. K. Howe C. J. (2010). Molecular basis of the waxy endosperm starch phenotype in broomcorn millet (*Panicum miliaceum* L.). *Mol. Biol. Evol.* 27 1478–1494. 10.1093/molbev/msq040 20139147 PMC2884200

[B54] IIMR, (2016). *Annual Report 2016-17.* Hyderabad: Indian Institute of Millets Research.

[B55] IIMR, (2018). *Annual Report 2017-18.* Hyderabad: Indian Institute of Millets Research.

[B56] Ishikawa G. Seimiya Y. Saito M. Nakamura T. Hoshino T. (2013). Molecular characterization of spontaneous and induced mutations in the three homoeologous waxy genes of Japanese barnyard millet [*Echinochloa esculenta* (A. Braun) H. Scholz]. *Mol. Breed.* 31 69–78. 10.1007/s11032-012-9769-9

[B57] Iwakami S. Uchino A. Kataoka Y. Shibaike H. Watanabe H. Inamura T. (2014). Cytochrome P450 genes induced by bispyribac-sodium treatment in a multiple-herbicide-resistant biotype of *Echinochloa phyllopogon*. *Pest Manag. Sci.* 70 549–558. 10.1002/ps.3572 23650123

[B58] Jagadish P. S. Mohapatra H. K. Chakravarthy M. K. Srivastava N. Nangia N. (2008). *A Compendium of Insect Pests of Finger Millet and Other Small Millets.* Available online at: http://www.aicrpsm.res.in/Downloads/Publications//A%20compendium%20of%20Insect%20pests%20of%20FM%20&%20other%20SM.pdf

[B59] Jain A. K. Jain S. K. Yadava H. S. (1997). Assessment of yield losses due to grain smut in barnyard millet. *Indian Phytopathol.* 50 49–52.

[B60] Jaiswal V. Gupta S. Gahlaut V. Muthamilarasan M. Bandyopadhyay T. Ramchiary N. (2019). Genome-wide association study of major agronomic traits in foxtail millet (*Setaria italica* L.) Using ddRAD sequencing. *Sci. Rep.* 9:5020. 10.1038/s41598-019-41602-6 30903013 PMC6430830

[B61] Jayaraman N. Suresh S. Nirmala Ganeshan N. M. (1997). “Genetic enhancement and breeding strategies in small millets,” in *Proceedings of the National Seminar on Small Millets: Current Research Trends and Future Priorities as Food Feed and in Processing for Value Addition*, Bangalore, 23–24.

[B62] Jaybhaye R. V. Srivastav P. P. (2015). Development of barnyard millet ready-to-eat snack food: Part II. *Food Sci. Res. J.* 6 285–291. 10.15740/HAS/FSRJ/6.2/285-291

[B63] Jia G. Huang X. Zhi H. Zhao Y. Zhao Q. Li W. (2013). A haplotype map of genomic variations and genome-wide association studies of agronomic traits in foxtail millet (*Setaria italica*). *Nat. Genet.* 45 957–961. 10.1038/ng.2673 23793027

[B64] Jia X. Zhang Z. Liu Y. Zhang C. Shi Y. Song Y. (2009). Development and genetic mapping of SSR markers in foxtail millet [*Setaria italica* (L.) P. Beauv.]. *Theor. Appl. Genet.* 118 821–829. 10.1007/s00122-008-0942-9 19139840

[B65] Johnson M. Deshpande S. Vetriventhan M. Upadhyaya H. D. Wallace J. G. (2019). Genome-Wide population structure analyses of three minor millets: kodo millet, little millet, and proso millet. *Plant Genome* 12:190021 10.3835/plantgenome2019.03.0021 PMC12810065 33016596

[B66] Joshi R. P. Jain A. K. Chauhan S. S. Singh G. (2015). Characterization of barnyard millet (Echinochloa frumentacea (Roxb.) Link.) landraces for agro-morphological traits and disease resistance. *Electron. J. Plant Breed.* 6 888–898.

[B67] Joshi V. (2013). Assessment of genetic variability and identification of genotypes for different traits in Barnyard millet (*Echinochola* spp.). *Int*. *J. Agric. Food Sci. Technol.* 4 65–67.

[B68] Kannan S. M. Thooyavathy R. A. Kariyapa R. T. Subramanian K. Vijayalakshmi K. (2013). *Data from: Seed Production Techniques for Cereals and Millets. Seed node of the Revitalizing Rainfed Agriculture Network Centre for Indian Knowledge Systems (CIICS).* Available at: http://www.ciks.org/downloads/seeds/5.%20Seed%20Production%20Techniques%20for%20Cereals%20and%20Millets.pdf (Accessed August 09, 2019).

[B69] Kaya H. Demirci M. Tanyolac B. (2014). Genetic structure and diversity analysis revealed by AFLP on different *Echinochloa* spp. from northwest Turkey. *Plant Syst. Evol.* 300 1–11. 10.1007/s00606-013-0965-9

[B70] Kim C. S. Alamgir K. M. Matsumoto S. Tebayashi S. I. Koh H. S. (2008). Antifeedants of Indian barnyard millet, *Echinochloa frumentacea* link, against brown planthopper, *Nilaparvata lugens* (Stål). *Sect. C J. Biosci.* 63 755–760. 10.1515/znc-2008-9-1022 19040117

[B71] Kim J. Y. Chang J. K. Park B.-R. Han S.-I. Choi K.-J. Kim S.-Y. (2011). Physicochemical and antioxidative properties of selected barnyard millet (*Echinochloa utilis*) species in Korea. *Food Sci. Biotechnol.* 20 461–469. 10.1007/s10068-011-0064-z

[B72] Kovach D. A. Widrlechner M. Brenner D. (2010). Variation in seed dormancy in Echinochloa and the development of a standard protocol for germination testing. *Seed Sci. Technol.* 38 559–571. 10.15258/sst.2010.38.3.04

[B73] Kraehmer H. Jabran K. Mennan H. Chauhan B. (2015). Global distribution of rice weeds - A review. *Crop Prot.* 80 73–86. 10.1016/j.cropro.2015.10.027

[B74] Krishna T. P. A. Maharajan T. David R. H. A. Ramakrishnan M. S. Ceasar A. Duraipandiyan V. (2018). Microsatellite markers of finger millet (*Eleusine coracana* (L.) Gaertn) and foxtail millet (*Setaria italica* (L.) Beauv) provide resources for cross-genome transferability and genetic diversity analyses in other millets. *Biocat. Agrl. Biotechnol.* 16 493–450.

[B75] Kumar A. Yadav S. Panwar P. Gaur V. Sood S. (2015). Identification of anchored simple sequence repeat markers associated with calcium content in finger millet (*Eleusine coracana*). *Proc. Natl. Acad. Sci. India B Biol. Sci.* 85 311–317. 10.1007/s40011-013-0296-1

[B76] Kumar L. D. Siva Sankar S. Venkatesh P. Hepcy Kalarani D. (2016). Green synthesis of silver nanoparticles using aerial parts extract of *Echinochloa colona* and their characterization. *Eur. J. Pharm. Med. Res.* 3 325–328.

[B77] Kumari K. Muthamilarasan M. Misra G. Gupta S. Alagesan S. Parida S. (2013). Development of eSSR-Markers in *Setaria italica* and their applicability in studying genetic diversity, cross-transferability and comparative mapping in millet and non-millet species. *PLoS One* 8:e67742. 10.1371/journal.pone.0067742 23805325 PMC3689721

[B78] Kumari K. S. Thayumanavan B. (1998). Characterization of starches of proso, foxtail, barnyard, kodo, and little millets. *Plant Foods Hum. Nutr.* 53:47 10.1023/A:1008083020810 10890757

[B79] Kuraloviya M. Vanniarajan C. Vetriventhan M. Babu C. Kanchana S. Sudhagar R. (2019). Qualitative characterization and clustering of early-maturing barnyard millet (*Echinochloa* spp.) germplasm. *Electron. J. Plant Breed.* 10:535 10.5958/0975-928x.2019.00067.x

[B80] Lata C. Prasad M. (2012). Validation of an allele-specific marker associated with dehydration stress tolerance in a core set of foxtail millet accessions. *Plant Breed.* 132 496–499. 10.1111/j.1439-0523.2012.01983.x

[B81] Lee J. Kim C.-S. Lee I.-Y. (2017). Discrimination of *Echinochloa colona* (L.) Link from other Echinochloa Species using DNA Barcode. *Weed Turfgrass Sci.* 4 225–229. 10.5660/WTS.2015.4.3.225

[B82] Lee J. Park K. Lee I. Kim C. Kown O. Park T. (2014). Simple sequence repeat analysis of genetic diversity among Acetyl-CoA carboxylase inhibitor-resistant and -susceptible Echinochloa crus-galli and *E. oryzicola* populations in Korea. *Weed Res.* 55 90–100. 10.1111/wre.12119

[B83] Li G. Wu S. Cai L. Wang Q. Zhao X. Wu C. (2013a). Identification and mRNA expression profile of glutamate receptor-like gene in quinclorac-resistant and susceptible Echinochloa crus-galli. *Gene* 531 489–495. 10.1016/j.gene.2013.09.013 24036427

[B84] Li G. Wu S. G. Yu R. X. Cang T. Chen L. P. Zhao X. P. (2013b). Identification and expression pattern of a glutathione S-transferase in *Echinochloa crus*-galli. *Weed Res.* 53 314–321. 10.1111/wre.12031

[B85] Lin H.-S. Chiang C. Y. Chang S.-B. Kuoh C.-S. (2011). Development of simple sequence repeats (SSR) Markers in *Setaria italica* (Poaceae) and cross-amplification in related species. *Int. J. Mol. Sci.* 12 7835–7845. 10.3390/ijms12117835 22174636 PMC3233442

[B86] Lohani U. Pandey J. Shahi N. (2012). Effect of degree of polishing on milling characteristics and proximate compositions of barnyard millet (*Echinochloa frumentacea*). *Food Bioprocess Technol.* 5 1113–1119. 10.1007/s11947-011-0518-6

[B87] Lule D. de Villiers S. Fetene M. Odeny D. A. Rathore A. Das R. R. (2018). Genetic diversity and association mapping of Ethiopian and exotic finger millet accessions. *Crop Pasture Sci.* 69 879–891. 10.1071/CP18175

[B88] Mace E. S. Tai S. Gilding E. K. Li Y. Prentis P. J. Bian L. (2013). Whole-genome sequencing reveals untapped genetic potential in Africa’s indigenous cereal crop sorghum. *Nat. Commun.* 4:2320. 10.1038/ncomms3320 23982223 PMC3759062

[B89] Manidool C. (1992). “Echinochloa crus-galli (L.) P. Beauv,” in *Plant Resources of South-East Asia*, eds t’ Mannetje Jones R. M., (Jodhpur: Pudoc Scientific Publishers), 303.

[B90] Manimekalai M. Dhasarathan M. Karthikeyan A. Murukarthick J. Renganathan V. G. Thangaraj K. (2018). Genetic diversity in the barnyard millet (Echinochola frumentacea) germplasms revealed by morphological traits and simple sequence repeat markers. *Curr. Plant Biol.* 14 71–78. 10.1016/j.cpb.2018.09.006

[B91] Maun M. A. Barrett S. C. (1986). The biology of Canadian weeds, *Echinochloa crus- galli* (L.) Beauv. *Can. J. Plant Sci.* 66 739–759. 10.4141/cjps86-093

[B92] Mauro-Herrera M. Wang X. Barbier H. Brutnell T. P. Devos K. M. Doust A. N. (2013). Genetic Control and Comparative Genomic Analysis of Flowering Time in Setaria (Poaceae). *G*3 3 283L–295L. 10.1534/g3.112.005207 23390604 PMC3564988

[B93] Mehta H. Tyagi P. C. Mohapatra K. P. (2005). Genetic diversity in Barnyard millet (*Echinochloa frumentacea* Roxb.). *Indian J. Genet.* 65 293–295.

[B94] Mishra A. K. Puranik S. Bahadur R. P. Prasad M. (2012). The DNA-binding activity of an AP2 protein is involved in transcriptional regulation of a stress-responsive gene, SiWD40, in foxtail millet. *Genomics* 100 252–263. 10.1016/j.ygeno.2012.06.012 22771384

[B95] Mitich L. W. (1990). Barnyardgrass. *Weed Technol.* 4 918–920.

[B96] Monteiro P. V. Sudharshana L. Ramachandra G. (1988). Japanese barnyard millet (*Echinochloa frumentacea*): protein content, quality and SDS-PAGE of protein fractions. *J. Sci. Food Agric.* 43:17e25.

[B97] Moreno Amador M. de Comino I. Sousa C. (2014). Alternative grains as potential raw material for gluten-free food development in the diet of celiac and gluten-sensitive patients. *Austin J. Nutr. Food Sci.* 2:9.

[B98] Mosovska S. Mikulasova M. Brindzova L. Valik L. Mikusova L. (2010). Genotoxic and antimutagenic activities of extracts from pseudocereals in the *Salmonella* mutagenicity assay. *Food Chem. Toxicol.* 48 1483–1487. 10.1016/j.fct.2010.03.015 20303377

[B99] Muldoon D. K. (1985). The effect of photoperiod on the growth and development of *Echinochloa* spp. Millets. *Aust. J. Exp. Agric*. 25, 428–433. 10.1071/EA9850428

[B100] Muldoon K. D. Pearson C. J. Wheeler J. (1982). The effect of temperature on growth and development of *Echinochloa* millets. *Ann. Bot.* 50 665–672. 10.1093/oxfordjournals.aob.a086408

[B101] Murukarthick J. Manimekalai M. Karthikeyan A. Perumal S. Dhasarathan M. Kandasamy T. (2019). Transcriptomes of Indian barnyard millet and barnyardgrass reveal putative genes involved in drought adaptation and micronutrient accumulation. *Acta Physiol. Plant.* 41:66 10.1007/s11738-019-2855-4

[B102] Muthamilarasan M. Prasad M. (2014). Advances in Setaria genomics for genetic improvement of cereals and bioenergy grasses. *Theor. Appl. Genet.* 128 1–14. 10.1007/s00122-014-2399-3 25239219

[B103] Muza F. R. Lee D. J. Andrews D. J. Gupta S. C. (1995). Mitochondrial DNA variation in finger millet [*Eleusine coracana* (L.) Gaertn.]. *Euphytica* 81 199–205. 10.1007/BF00025434

[B104] Nagaraja A. Mantur S. G. (2008). Evaluation of barnyard millet entries for grain smut resistance and yield. *Mysore J. Agric. Sci.* 42 375–377.

[B105] Nah G. Im J.-H. Kim J.-W. Park H.-R. Yook M.-J. Yang T.-J. (2015). Uncovering the differential molecular basis of adaptive diversity in three echinochloa leaf transcriptomes. *PLoS One* 10:e0134419. 10.1371/journal.pone.0134419 26266806 PMC4534374

[B106] Nandini R. Fakrudin B. (1999). Emasculation in finger millet using hot water. *Mysore J. Agric. Sci.* 33 6–10.

[B107] Nazni P. Shobana D. R. (2016). Effect of processing on the characteristics changes in barnyard and foxtail millet. *J. Food Process. Technol.* 7:566 10.4172/2157-7110.1000566

[B108] Nguyen H. D. Bingtian Z. Le D. A. T. Yoon Y. H. Ko J. Y. Woo K. S. (2016). Isolation of lignan and fatty acid derivatives from the grains of *Echinochloa utilis* and Their Inhibition of Lipopolysaccharide-Induced Nitric Oxide Production in RAW 264.7 cells. *J. Agric. Food Chem.* 64 425–432. 10.1021/acs.jafc.5b05638 26725284

[B109] Nirgude M. Babu B. K. Shambhavi Y. Singh U. M. Upadhyaya H. D. Kumar A. (2014). Development and molecular characterization of genic molecular markers for grain protein and calcium content in finger millet (*Eleusine coracana* (L.) Gaertn.). *Mol. Biol. Rep.* 41 1189–1200. 10.1007/s11033-013-2825-7 24477581

[B110] Nirmalakumari A. Vetriventhan M. (2009). Phenotypic analysis of anther and pollen in diversified genotype of barnyard millet (*Echinochloa frumentaceae*) floral characters. *ICFAI Univ. J. Genet. Evol.* 2 12–16.

[B111] Nolde S. Vassilevski A. A. Rogozhin E. A. Barinov N. A. Balashova T. A. Samsonova O. V. (2011). Disulfide-stabilized helical hairpin structure and activity of a novel antifungal peptide EcAMP1 from seeds of barnyard grass (*Echinochloa crus*-galli). *J. Biol. Chem.* 286 25145–25153. 10.1074/jbc.M110.200378 21561864 PMC3137087

[B112] Nozawa S. Takahashi M. Nakai H. Sato Y.-I. (2006). Difference in SSR Variations between Japanese Barnyard Millet (*Echinochloa esculenta*) and its Wild Relative *E. crus-galli*. *Breed. Sci.* 56 335–340. 10.1270/jsbbs.56.335 26081539

[B113] Obidiegwu O. N. Obidiegwu J. E. Parzies H. (2013). Development of SSR for foxtail millet (*Setaria italica* (L.) P. Beauv.) and its utility in genetic discrimination of a core set. *Genes Genomics* 35 609–615. 10.1007/s13258-013-0110-8

[B114] Odintsova T. I. Rogozhin E. A. Baranov Y. Musolyamov A. K. Yalpani N. Egorov T. A. (2008). Seed defensins of barnyard grass *Echinochloa crusgalli* (L.) Beauv. *Biochimie* 90 1667–1673. 10.1016/j.biochi.2008.06.007 18625284

[B115] Okada M. Hanson B. D. Hembree K. J. Peng Y. Shrestha A. Wright D. (2013). Evolution and spread of glyphosate resistance in *Conyza canadensis* in California. *Evol. Appl.* 6 761–777. 10.1111/eva.12061 29387164 PMC5779124

[B116] Olanrewaju O. S. Glick B. R. Babalola O. O. (2017). Mechanisms of action of plant growth promoting bacteria. *World J. Microbiol. Biotechnol.* 33:197. 10.1007/s11274-017-2364-9 28986676 PMC5686270

[B117] Osuna M. Okada M. Ahmad R. Fischer A. Jasieniuk M. (2011). Genetic Diversity and spread of thiobencarb resistant early watergrass (*Echinochloa oryzoides*) in California. *Weed Sci.* 59 195–201. 10.1614/WS-D-10-00124.1

[B118] Padulosi S. Bhag M. C. Bala R. S. Gowda J. V. Gowda K. T. K. Shanthakumar G. C. (2009). Food security and climate change: role of plant genetic resources of minor millets. *Indian J. Plant Genet. Resour.* 22 1–16.

[B119] Pandey G. Misra G. Kumari K. Gupta S. Kumar Parida S. Chattopadhyay D. (2013). Genome-wide development and use of microsatellite markers for large-scale genotyping applications in foxtail millet [*Setaria italica* (L.)]. *DNA Res.* 20 197–207. 10.1093/dnares/dst002 23382459 PMC3628449

[B120] Panwar P. Dubey A. Verma A. K. (2016). Evaluation of nutraceutical and antinutritional properties in barnyard and finger millet varieties grown in Himalayan region. *J. Food Sci. Technol.* 53 2779e2787. 10.1007/s13197-016-2250-8 PMC4951431 27478234

[B121] Perumal S. Jayakodi M. Kim D.-S. Yang T.-J. Natesan S. (2016). The complete chloroplast genome sequence of Indian barnyard millet, *Echinochloa frumentacea* (Poaceae). *Mitochondrial DNA* 1 79–80. 10.1080/23802359.2015.1137832 PMC7871652 33644328

[B122] Prabha D. Negi Y. K. Khanna V. K. (2010). Morphological and isozyme diversity in the accessions of two cultivated species of barnyard millet. *Nat. Sci.* 8 71–76.

[B123] Prabha D. Negi Y. K. Khanna V. K. (2012). Assessment of genetic diversity of barnyard millet accessions using molecular markers. *Indian J. Plant Genet. Resour.* 25 174–179.

[B124] Prakash R. Vanniarajan C. (2013). Genetic variability for panicle characters in indigenous and exotic barnyard millet (*Echinochloa frumentacea* (Roxb.) Link) germplasm over environment. *Veg. An Int. J. Plant Res.* 26:297 10.5958/j.2229-4473.26.2.088

[B125] Qie L. Jia G. Zhang W. Schnable J. Shang Z. Li W. (2014). Mapping of Quantitative Trait Locus (QTLs) that contribute to germination and early seedling drought tolerance in the interspecific cross *Setaria italica*×*Setaria viridis*. *PLoS One* 9:e101868. 10.1371/journal.pone.0101868 25033201 PMC4102488

[B126] Radhamani J. Pandey A. Srinivasan K. Tyagi V. (2011). Conserving millet genetic resources in India. *Proc. Indian Natl. Sci. Acad.* 77 295–304.

[B127] Raj S. M. Mahudeswaran K. Shanmugasundaran A. (1964). Observation on the hot water technique of emasculation of ragi flowers (*Eleusine coracana* (L.) Gaertn.). *Madras Agric. J.* 51 71–75.

[B128] Rajput S. G. Santra D. K. (2016). Evaluation of genetic diversity of proso millet germplasm available in the United States using simple-sequence repeat markers. *Crop Sci.* 56 2401–2409. 10.2135/cropsci2015.10.0644

[B129] Rajput S. G. Santra D. K. Schnable J. (2016). Mapping QTLs for morpho-agronomic traits in proso millet (*Panicum miliaceum* L.). *Mol. Breed.* 36:37 10.1007/s11032-016-0460-4

[B130] Ramakrishnan M. Antony Ceasar S. Duraipandiyan V. Al-Dhabi N. A. Ignacimuthu S. (2016). Assessment of genetic diversity, population structure and relationships in Indian and non-Indian genotypes of finger millet (*Eleusine coracana* (L.) Gaertn) using genomic SSR markers. *Springerplus* 5 1–11. 10.1186/s40064-015-1626-y 26900542 PMC4749518

[B131] Ramakrishnan M. Ceasar S. A. Vinod K. K. Duraipandiyan V. Ajeesh Krishna T. P. Upadhyaya H. D. (2017). Identification of putative QTLs for seedling stage phosphorus starvation response in finger millet (*Eleusine coracana* L. Gaertn.) by association mapping and cross species synteny analysis. *PLoS One* 12:e0183261. 10.1371/journal.pone.0183261 28820887 PMC5562303

[B132] Rawat L. Nautiyal A. Bisht T. S. Prasad S. Naithani D. Makhloga K. (2019). Screening of barnyard millet germplasm against shoot fly and stem borer damage under field conditions. *Int. J. Curr. Microbiol. Appl. Sci.* 8 1221–1226. 10.20546/ijcmas.2019.802.142

[B133] Renganathan V. G. Ibrahim S. M. Vanniarajan C. (2015). Combining ability for quantitative traits in Barnyard millet (*Echinochloa frumentaceae* (Roxb.) Link). *Electron. J. Plant Breed.* 6 389–394.

[B134] Renganathan V. G. Vanniarajan C. Nirmalakumari A. Raveendran M. Thiyageshwari S. (2017). Cluster analyses for qualitative and quantitative traits in barnyard millet *Echinochloa frumentacea* (Roxb.Link) germplasm. *Bioscan* 12 1927–1931.

[B135] Renganathan V. G. Vanniarajan C. Ramalingam J. (2019). “Genetic analysis and identification of molecular markers linked to the anthocyanin pigmentation in barnyard millet [*Echinochloa frumentacea* Roxb (Link)],” in *Proceedings of the Neglected and Underutilized crop species for Food, Nutrition, Energy and Environment*, (New Delhi: NIPGR), 43.

[B136] Ruiz-Santaella P. J. Bastida F. Franco A. Prado R. (2006). Morphological and molecular characterization of different *Echinochloa* spp. and *Oryza sativa* populations. *J. Agric. Food Chem.* 54 1166–1172. 10.1021/jf0520746 16478232

[B137] Rutledge J. Talbert R. E. Sneller C. H. (2000). RAPD analysis of genetic variation among propanil-resistant and -susceptible Echinochloa crus-galli populations in Arkansas. *Weed Sci.* 48 669–674. 10.1614/0043-1745(2000)048[0669:raogva]2.0.co;2

[B138] Saleh A. Zhang Q. Chen J. Shen Q. (2013). Millet grains: nutritional quality, processing, and potential health benefits. *Compr. Rev. Food Sci. Food Saf.* 12 281–295. 10.1111/1541-4337.12012

[B139] Santra D. Heyduck R. Baltensperger D. Graybosch R. Nelson L. Frickel G. (2015). Registration of ‘Plateau’ Waxy (Amylose-Free) proso millet. *J. Plant Regist.* 9:41 10.3198/jpr2013.11.0067crc

[B140] Sato K. Mukainari Y. Naito K. Fukunaga K. (2013). Construction of a foxtail millet linkage map and mapping of spikelet-tipped bristles1 (stb1) by using transposon display markers and simple sequence repeat markers with genome sequence information. *Mol. Breed.* 31 675–684. 10.1007/s11032-012-9825-5

[B141] Sayani R. Chatterjee A. (2017). Nutritional and biological importance of the weed *Echinochloa colona*: a review. *Int. J. of Food Sci. Biotechnol.* 2 31–37. 10.11648/j.ijfsb.20170202.13

[B142] Sebastin R. Lee K. J. Cho G. T. Lee J. R. Shin M. J. Kim S. H. (2019). The complete chloroplast genome sequence of Japanese Millet *Echinochloa esculenta* (A. braun) H. scholz (Poaceae). *Mitochondrial DNA Part B Resour.* 4 1392–1393. 10.1080/23802359.2019.1598787

[B143] Seetharam A. Gowda J. Halaswamy J. H. (2003). “Small millets,” in *Nucleus and Breeder Seed Production Manual*, eds Chowdhury S. K. Lal S. K., (New Delhi: Indian Agricultural Research Institute), 54–67.

[B144] Sharma A. Sood S. Agrawal P. K. Kant L. Bhatt J. C. Pattanayak A. (2016). Detection and assessment of nutraceuticals in methanolic extract of finger (*Eleusine coracana*) and Barnyard Millet (*Echinochloa frumentacea*). *Asian J. Chem.* 28 1633–1637. 10.14233/ajchem.2016.19790

[B145] Sharma D. Tiwari A. Sood S. Jamra G. Singh N. K. Meher P. K. (2018). Genome wide association mapping of agro-morphological traits among a diverse collection of finger millet (*Eleusine coracana* L.) genotypes using SNP markers. *PLoS One* 13:e19444. 10.1371/journal.pone.0199444 30092057 PMC6084814

[B146] Siles M. Baltensperger D. Nelson L. A. (2001). Technique for Artificial Hybridization of Foxtail Millet [(L.) Beauv.]. *Crop Sci.* 41 1408–1412. 10.2135/cropsci2001.4151408x

[B147] Singh H. S. Singh K. (2005). “Status and needs of pasture and fodder management in Uttaranchal,” in *Road Map for Pasture and Fodder Development in NWHR for Livestock Sustenance*, eds Bisht J. K. Srivastava A. K., (Almora: Vivekananda Parvatiya Krishi Anusandhan Sansthan), 39–64.

[B148] Singh K. P. Mishra H. N. Saha S. (2010). Moisture-dependent properties of barnyard millet grain and kernel. *J. Food Eng.* 96 598–606. 10.1016/j.jfoodeng.2009.09.007

[B149] Song B. Y. Shi J. X. Song S. Q. (2015). Dormancy release and germination of *Echinochloa crus-galli* grains in relation to galactomannan-hydrolysing enzyme activity. *J. Integr. Agric.* 14 1627–1636. 10.1016/S2095-3119(14)60940-0

[B150] Sood S. Khulbe R. Kumar R. A. Agrawal P. K. Upadhyaya H. (2015). Barnyard millet global core collection evaluation in the sub mountain Himalayan region of India using multivariate analysis. *Crop J.* 3 517–525. 10.1016/j.cj.2015.07.005

[B151] Sood S. Khulbe R. Saini N. Gupta A. Agrawal P. K. (2014). Research Note Interspecific Hybrid between Echinochloa esculenta (Japanese barnyard millet) and E. frumentacea (Indian barnyard millet) – A New Avenue for Genetic Enhancement of Barnyard Millet. *Electron. J. Plant Breed.* 5 248–253.

[B152] Sridevi R. Manonmani V. (2016). Seed priming effect on physiological traits of kodo millet and barnyard millet. *Int. J. Agric. Sci. Res.* 6 187–194.

[B153] Sridhar R. Lakshminarayana G. (1992). Lipid class contents and fatty acid composition of small millets: barnyard little (*Panicum sumatrense*), kodo (*Paspalum scrobiculatum*), and barnyard (*Echinochloa colona*). *J. Agric. Food Chem.* 40:2131e2134.

[B154] Subhashini V. Swamy A. V. V. S. (2014). Screening Potential of three native grass species for phyto remediation of heavy metals. *Int. J. Eur. Acad. Res.* 4 5887–5903.

[B155] Sujatha K. Selvarani K. Vijayalakshmi V. Vanniarajan C. Sivasubramaniam K. (2013). Seed fortification studies in barnyard millet (*Echinochloa frumentacea*) cv. CO1. *IOSR J. Agric. Vet. Sci.* 5 22–24. 10.9790/2380-0542224

[B156] Sun J. Luu N. S. Chen Z. Chen B. Cui X. Wu J. (2019). Generation and Characterization of a Foxtail Millet (*Setaria italica*) Mutant Library. *Front. Plant Sci.* 10:369. 10.3389/fpls.2019.00369 31001298 PMC6455083

[B157] Sundararaj D. P. Thulasidas G. (1976). *Botany of Field Crops.* New Delhi: Macmillan Publisher.

[B158] Sung S.-J. Leather G. R. Hale M. G. (1987). Development and germination of barnyardgrass (*Echinochloa crus-galli*) seeds. *Weed Sci.* 35 211–215. 10.1017/S0043174500079078

[B159] Surekha N. Ravikumar S. N. Mythri S. Rohini D. (2013). Barnyard Millet (*Echinochloa frumentacea* Link) cookies: development, value addition, consumer acceptability, nutritional and shelf life evaluation. *IOSR J. Environ. Sci. Toxicol. Food Technol.* 7 01–10. 10.9790/2402-0730110

[B160] Suryawanshi V. Talke I. N. Weber M. Eils R. Brors B. Clemens S. (2016). Between-species differences in gene copy number are enriched among functions critical for adaptive evolution in *Arabidopsis halleri*. *BMC Genomics* 17:1034. 10.1186/s12864-016-3319-5 28155655 PMC5259951

[B161] Tabacchi M. Mantegazza R. Spada A. Ferrero A. (2009). Morphological traits and molecular markers for classification of *Echinochloa* species from Italian rice fields. *Weed Sci.* 54 1086–1093. 10.1614/WS-06-018R1.1

[B162] Trivedi A. K. Arya L. Verma S. Tyagi R. Hemantaranjan A. (2017). Evaluation of barnyard millet diversity in central Himalayan region for environmental stress tolerance. *J. Agric. Sci.* 155 1–11. 10.1017/S0021859617000545

[B163] Ugare R. Chimmad B. Naik R. Bharati P. Itagi S. (2014). Glycemic index and significance of barnyard millet (*Echinochloa frumentacae*) in type II diabetics. *J. Food Sci. Technol.* 51 392–395. 10.1007/s13197-011-0516-8 24493902 PMC3907638

[B164] Upadhyaya H. Dwivedi S. L. Singh S. K. Sube S. Vetriventhan M. Sharma S. (2014). Forming core collections in Barnyard, Kodo, and little millets using morphoagronomic descriptors. *Crop Sci.* 54 2673–2682. 10.2135/cropsci2014.03.0221

[B165] Upadhyaya H. Gowda C. Sastry D. (2008). Plant genetic resources management: collection, characterization, conservation and utilization. *J. SAT Agric. Res.* 6:16.

[B166] Vanniarajan C. Anand G. Kanchana S. Arun Giridhari V. Renganathan V. G. (2018). A short duration high yielding culture - Barnyard millet ACM 10145. *Agric. Sci. Dig. A Res. J.* 8 123–126. 10.18805/ag.D-4574

[B167] Varshney R. K. Hoisington D. A. Tyagi A. K. (2006). Advances in cereal genomics and applications in crop breeding. *Trends Biotechnol.* 24 490–499. 10.1016/j.tibtech.2006.08.006 16956681

[B168] Varshney R. K. Shi C. Thudi M. Mariac C. Wallace J. Qi P. (2017). Pearl millet genome sequence provides a resource to improve agronomic traits in arid environments. *Nat. Biotechnol.* 35 969–976. 10.1038/nbt.3943 28922347 PMC6871012

[B169] Veena B. Chimmad B. V. Naik R. K. Shantakumar G. (2004). Development of barnyard millet based traditional foods. *Karnataka J. Agric. Sci*. 17, 522–527.

[B170] Veena S. Chimmad B. V. Naik R. K. Shanthakumar G. (2005). Physico-chemical and nutritional studies in Barnyard millet. *Karnataka J. Agric. Sci.* 18:101e105.

[B171] Veeranagamallaiah G. Puli C. Jyothsnakumari G. Sudhakar C. (2007). Glutamine synthetase expression and pyrroline-5-carboxylate reductase activity influence proline accumulation in two cultivars of foxtail millet (*Setaria italica* L.) with differential salt sensitivity. *Environ. Exp. Bot.* 60 239–244. 10.1016/j.envexpbot.2006.10.012

[B172] Vijayakumar T. P. Mohankumar J. B. Jaganmohan R. (2009). Quality evaluation of chapati from millet flour blend incorporated composite flour. *Indian J. Nutr. Diet.* 46 144–155.

[B173] Wallace G. J. Upadhyaya H. Vetriventhan M. Buckler E. Hash Charles J. Ramu P. (2015). The genetic makeup of a global barnyard millet germplasm collection. *Plant Genome* 08 01–07. 10.3835/plantgenome2014.10.0067 33228281

[B174] Wang J. Wang Z. Du X. Yang H. Han F. Han Y. (2017). A high-density genetic map and QTL analysis of agronomic traits in foxtail millet [*Setaria italica* (L.) P. Beauv.] using RAD-seq. *PLoS One* 12:e0179717. 10.1371/journal.pone.0179717 28644843 PMC5482450

[B175] Wang J. Wang Z. L. Yang H. Q. Yuan F. Guo E. H. Tian G. (2013). Genetic analysis and preliminary mapping of a highly male-sterile gene in foxtail millet (*Setaria italica* L. Beauv.) using SSR markers. *J. Integr. Agric.* 12 2143–2148. 10.1016/S2095-3119(13)60392-5

[B176] Wanous M. K. (1990). Origin, taxonomy and ploidy of the millets and minor cereals. *Plant Var. Seeds* 3 99–112.

[B177] Weber A. (2015). Discovering new biology through RNA-Seq. *Plant Physiol.* 169:01081.2015. 10.1104/pp.15.01081 26353759 PMC4634082

[B178] Wendel J. F. (2015). The wondrous cycles of polyploidy in plants. *Am. J. Bot.* 102 1753–1756. 10.3732/ajb.1500320 26451037

[B179] Xu W. Di C. Zhou S. Liu J. Li L. Liu F. (2015). Rice transcriptome analysis to identify possible herbicide quinclorac detoxification genes. *Front. Genet.* 6:306. 10.3389/fgene.2015.00306 26483837 PMC4586585

[B180] Yabuno T. (1962). Cytotaxonomic studies on the two cultivated species and the wild relatives in the genus *Echinochloa*. *Cytologia* 27 296–305. 10.1508/cytologia.27.296 25058746

[B181] Yabuno T. (1966). Biosystematic study of the genus *Echinochloa*. *Jpn. J. Bot.* 19 277–323.

[B182] Yabuno T. (1984). A biosystematic study on *Echinochloa oryzoides* (Ard.) Fritsch. *Cytologia* 49 673–678. 10.1508/cytologia.49.673 25058746

[B183] Yabuno T. (1987). Japanese barnyard millet (*Echinochloa utilis*. Poaceae) in Japan. *Econ. Bot.* 41 484–493. 10.1007/BF02908141

[B184] Yabuno T. (1996). “Taxonomy and phylogeny of the genus *Echinochloa*,” in *Natural History of Genus Echinochloa*, eds Yabuno T. Yamaguchi H., (Tokyo: Daw Elanco/Dow Chemical Company), 16–28.

[B185] Yabuno T. (2001). “Taxonomy and phylogeny of the genus *Echinochloa*,” in *Natural History of Genus Echinochloa*, eds Yabuno T. Yamaguchi H., (Tokyo: Zennokyo Shuppan), 15–30.

[B186] Yadav R. Yadav V. (2013). Comparative performance of Indian and Japanese barnyard millet cultivars under varied fertility conditions for dual use in Indian Central Himalaya. *Range Manag. Agrofor.* 34 175–178.

[B187] Yadav S. Gaur V. Jaiswal J. P. Kumar A. (2014). Simple sequence repeat (SSR) analysis in relation to calcium transport and signaling genes reveals transferability among grasses and a conserved behavior within finger millet genotypes. *Plant Syst. Evol.* 300 1–8. 10.1007/s00606-014-0982-3

[B188] Yamaguchi H. Utano A. Y. A. Yasuda K. Yano A. Soejima A. (2005). A molecular phylogeny of wild and cultivated *Echinochloa* in East Asia inferred from non-coding region sequences of trnT-L-F. *Weed Biol. Manag.* 5 210–218. 10.1111/j.1445-6664.2005.00185.x

[B189] Yang X. Yu X.-Y. Li Y. F. (2013). De novo assembly and characterization of the barnyardgrass (*Echinochloa crus-galli*) transcriptome using next-generation pyrosequencing. *PLoS One* 8:e69168. 10.1371/journal.pone.0069168 23874903 PMC3707877

[B190] Yasuda K. Yano A. Nakayama Y. Yamaguchi H. (2006). Molecular identification of *Echinochloa oryzicola* Vasing. and *E. crus-galli* (L.) Beauv. using a polymerase chain reaction–restriction fragment length polymorphism technique. *Weed Biol. Manag.* 2 11–17. 10.1046/j.1445-6664.2002.00041.x

[B191] Ye C.-Y. Lin Z. Li G. Wang Y. Qiu J. Fu F. (2014). Echinochloa chloroplast genomes: insights into the evolution and taxonomic identification of two weedy species. *PLoS One* 9:e113657. 10.1371/journal.pone.0113657 25427255 PMC4245208

[B192] Yoshitsu Y. Takakusagi M. Abe A. Takagi H. Uemura A. Yaegashi H. (2017). QTL-seq analysis identifies two genomic regions determining the heading date of foxtail millet. *Setaria italica* (L.) P.Beauv. *Breed. Sci.* 67 518–527. 10.1270/jsbbs.17061 29398946 PMC5790050

[B193] Yuichiro M. Shinya U. Yamaguchi H. (1999). Identification of polyploid groups in the genus Echinochloa by isozyme analysis. *J. Weed Sci. Technol.* 44 205–217. 10.3719/weed.44.205

[B194] Zee S. Y. O’brien T. P. (1971). Aleurone Transfer Cells and other Structural Features of the Spikelet of Millet. *Aust. J. Biol. Sci.* 24 391–396.

[B195] Zegada-Lizarazu W. Iijima M. (2005). Deep root water uptake ability and water use efficiency of pearl millet in comparison to other millet species. *Plant Prod. Sci.* 8 454–460. 10.1626/pps.8.454

[B196] Zhang G. Liu X. Quan Z. Cheng S. Xu X. Pan S. (2012). Genome sequence of foxtail millet (*Setaria italica*) provides insights into grass evolution and biofuel potential. *Nat. Biotechnol.* 30 549–554. 10.1038/nbt.2195 22580950

[B197] Zhang K. Fan G. Zhang X. Zhao F. Wei W. Du G. (2017). Identification of QTLs for 14 agronomically important traits in *Setaria italica* based on SNPs generated from high-throughput sequencing. *G3* 7 1587–1594. 10.1534/g3.117.041517 28364039 PMC5427501

[B198] Zhang S. Tang C. Zhao Q. Li J. Yang L. Qie L. (2014). Development of highly polymorphic simple sequence repeat markers using genome-wide microsatellite variant analysis in Foxtail millet [*Setaria italica* (L.) P. Beauv.]. *BMC Genomics* 15:78. 10.1186/1471-2164-15-78 24472631 PMC3930901

[B199] Zhao W. Lee G. A. Kwon S. W. Ma K. H. Lee M. C. Park Y. J. (2012). Development and use of novel SSR markers for molecular genetic diversity in Italian millet (*Setaria italica* L.). *Genes Genom.* 34 51–57. 10.1007/s13258-011-0102-5

[B200] Zou C. Li L. Miki D. Li D. Tang Q. Xiao L. (2019). The genome of broomcorn millet. *Nat. Commun.* 10:436. 10.1038/s41467-019-08409-5 30683860 PMC6347628

