# Supplementary material for: Barnyard Millet for Food and Nutritional Security: Current Status and Future Research Direction
Source: Front Genet. 2020 Jun 23;11:500. doi: 10.3389/fgene.2020.00500 (PMC7325689; doi:10.3389/fgene.2020.00500)
Supplement: Supplementary file 1 [file Table_1.DOC]

**Supplementary Table S1 Nutritional compositions of barnyard millet grains.**

| **Nutritional composition** | **Compounds** | **Content** |
| --- | --- | --- |
| Starch (g/100g) | Carbohydrate | 51.5-62.0 |
|  | Amylose (%) | 20.0 |
| Crude Protein (%) | Albumin | 11.3-17.2 |
|  | Globulins | 11.3-17.2 |
|  | Prolamins | 14.3-20.9 |
|  | Glutelins | 45.2-63.5 |
| Essential amino acids (mg/100g) | *Phenylalanine* | 5.5-6.3 |
|  | *Histidine* | 1.8-2.0 |
|  | *Isoleucine* | 4.5-4.6 |
|  | *Leucine* | 11.4-11.7 |
|  | *Lysine* | 1.6-1.8 |
|  | *Methionine* | 1.6-2.0 |
|  | *Threonine* | 3.6-3.7 |
|  | *Tryptophan* | 1.0 |
|  | *Valine* | 6.1-6.2 |
| Fat (g/100g) | Crude Fat | 2.5-6.3 |
| Ash (g/100g) | Ash | 4.7-5.0 |
| Fiber (%) | Crude fiber | 8.1-16.3 |
|  | Total Dietary Fiber | 23.3-31.7 |
|  | Insoluble Dietary Fiber | 22.0-26.0 |
|  | Soluble Dietary Fiber | 0.6-9.8 |
| Minerals (mg/100g) | Calcium | 22 |
|  | Iron | 18.6 |
|  | Zinc | 4.9 |
|  | Magnesium | 86.2 |
|  | Copper | 0.6 |
|  | Manganese | 0.7 |
| Vitamins (mg/100g) | Thiamin | 0.4 |
|  | Riboﬂavin | 0.1 |
|  | Niacin | 4.2 |
| Fatty Acid (%) | Palmitic | 17.4-19.0 |
|  | Palmitoleic | 0.4 |
|  | Stearic | 4.3-4.5 |
|  | Oleic | 27.6-28.2 |
|  | Linoleic | 46.4-48.1 |
|  | Linolenic | 1.5-1.7 |
| Lipids (%) | Total lipids | 8.0 |
|  | Total phospholipids | 5.5 |
| Phenolic compounds (mg/g) | Total phenols | 0.8 |
|  | Flavonoids | 0.6 |
| Carotenoids (mg/100g) | Total carotenoids | 36.7-50.8 |
| Bioactive compounds (g/100g) | GABA | 11.5-12.3 |
|  | *β*-glucan | 5.0-6.0 |
| Anti-nutritional factor (mg/g) | Phytic acid | 3.30-3.70 |

*Note: GABA, Gamma amino butyric acid; Sources, Monteiro et al., 1988; Sridhar and Lakshminarayana, 1992; Kumari and Thayumanavan, 1998; Veena et al., 2005; Saleh et al., 2013; Chandel et al., 2014; Ugare et al., 2014; Panwar et al., 2016; Sharma et al., 2016; Renganathan et al., 2017;*

**Supplementary Table S2 Variability in agronomic traits of barnyard millet accessions across India**.

| **Traits** | **Rewa, Madhya Pradesh^a^**  **(49 accessions)** | **Ranichauri, Uttarakhand^b^ (58 accessions)** | **‎Patancheru, Telangana^c^ (220 accessions)** | **Almora, Uttarakhand^d^ (95 accessions)** | **Central Himalayan Region,**  **Uttarakhand^e^**  **(178 accessions)** | **Hyderabad,**  **Andhra Pradesh^f^**  **(146 accessions)** | **Madurai, Tamil Nadu^g^**  **(40 accessions)** | **Overall range** |
| --- | --- | --- | --- | --- | --- | --- | --- | --- |
| Days to 50% flowering | 35.0-45.0 | 40.0-56.0 | 31.0–77.0 | 31.0-68.0 | 48.0-88.0 | 57.0-92.0 | NA | 27.0-92.0 |
| Plant height (cm) | 65.1-128.9 | 65.1-182.1 | 44.5–196.5 | 79.7-156.8 | 54.7-161.2 | 37.0-152.0 | 99.7-208.1 | 29.0-235.0 |
| Flag leaf length (cm) | 9.9-29.5 | 11.0-32.5 | 10.3–31.1 | 12.3-31.3 | 13.0-35.3 | 2.0-34.0 | 15.8-33.2 | 2.0-35.3 |
| Flag leaf breadth (cm) | 1.0-3.4 | 1.6-3.4 | 0.7–3.2 | 1.4-3.0 | 1.0-4.7 | 0.5-3.0 | 1.9-4.5 | 0.5-4.7 |
| Panicle length (cm) | 9.8-19.2 | 9.6-21.4 | 8.1–25.8 | 12.12-24.0 | 11.2-23.7 | 6.0-25 | 12.5-31.0 | 1.3-31.0 |
| No. of tillers | 1.0-4.6 | 1.8-15.2 | 3.9–20.1 | 1.2-9.3 | NA | 1.0-36.0 | 3.0-10.5 | 1.0-44.0 |
| No. of racemes/inflor. | NA | 5.7-44.3 | 21.9–30.4 | 8.7-49.7 | NA | 7.0-54.0 | 22.0-64.5 | 5.0-64.5 |
| Peduncle length (cm) | 2.6-10.0 | 3.4-41.5 | 6.9–27.7 | 8.1-29.8 | 2.0-12.4 | 5.0-20.0 | 1.7-11.3 | 1.7-41.5 |
| Culm thickness (cm) | NA | 0.6-1.3 | 0.5–0.7 | 0.3-0.9 | NA | NA | NA | 0.3-1.3 |
| Len. of lower raceme (cm) | 1.8-5.9 | NA | 2.6–3.9 | 1.8-6.2 | NA | 1.5-9.5 | 2.1-7.2 | 1.5-9.5 |
| 1000 grain weight (g) | 2.6-3.8 | NA | NA | NA | 2·0-5·8 | 2.0-2.17 | 2.4-3.9 | 2.0-3.9 |
| Days to maturity | NA | 73.0-90.0 | NA | 58.0-91.0 | 96.0-133.0 | NA | 78.0-108.0 | 58.0-133.0 |
| Grain yield per plant (g) | 2.9-13.2 | 0.4-26.8 | NA | 4.2–24.3 | 0·3-9·3 | 8.3-36.4 | 7.6-46.7 | 2.9-46.7 |

*Note: ^a^Joshi, 2013, ^b^Joshi et al., 2015, ^c^Upadhyaya et al., 2014, ^d^Sood et al., 2015*, *^e^Trivedi et al., 2017, ^f^Indian Institute of Millets Research, 2017,* *^g^Renganathan et al., 2017, NA, not available*

**Supplementary Table S3** Details on genomic resources available for cultivated small millets and its wild relatives.

| **Crop** | **Species** | **Genome size**  **(Gb)** | **No. of Genome assembly** | **Nucleotide sequence** | **GEO datasets** | **EST sequence** | **SRA** | **Gene sequence** | **Protein sequence** | **Protein structure** |
| --- | --- | --- | --- | --- | --- | --- | --- | --- | --- | --- |
| Barnyard millet | *Echinochloa crus-galli^a^* | 1.49 | 1 | 679 | NA | 74 | 48 | 170 | 985 | 1 |
|  | *Echinochloa colona^a^* | NA | NA | 190 | 1 | NA | 21 | 130 | 438 | NA |
|  | *Echinochloa oryzicola^a^* | NA | NA | 232 | NA | NA | 2 | 132 | 215 | NA |
|  | *Echinochloa frumentacea^b^* | NA | NA | 43 | NA | NA | 4 | 130 | 198 | NA |
|  | *Echinochloa esculenta^b^* | NA | NA | 65 | NA | NA | 22 | 130 | 200 | NA |
| Finger millet | *Eleusine indica^a^* | 0.49 | 1 | 679 | NA | 47 | 22 | 195 | 414 | NA |
|  | *Eleusine coracana^b^* | 1.21 | 1 | 1934 | 6 | 1934 | 237 | 195 | 559 | 3 |
| Foxtail millet | *Setaria viridis^a^* | 0.40 | 1 | NA | 204 | 1 | 1214 | 133 | 53073 | NA |
|  | *Setaria italica^b^* | 0.44 | 2 | 66027 | 20 | 66027 | 3029 | 32135 | 80105 | NA |
| Kodo millet | *Paspalum scrobiculatum^b^* | 1.9 | NA | 60 | NA | 29 | 1 | NA | 8 | NA |
| Little millet | *Panicum sumatrense^b^* | NA | 2 | 18 | 87 | NA | 3 | 132 | 181 | NA |
|  | *Panicum psilopodium^a^* | NA | NA | NA | NA | NA | NA | NA | 3 | NA |
| Proso millet | *Panicum miliaceum^b^* | 0.85 | 2 | 1085 | 87 | 195 | 137 | 135 | 56255 | NA |
|  | *Panicum repens^a^* | 1.2 | NA | 92 | NA | NA | 4 | NA | 55 | NA |
|  | *Panicum capillare^a^* | NA | NA | 65 | NA | NA | 25 | 131 | 217 | NA |

*Note: ^a^wild relatives/progenitor, ^b^cultivated species, NA, not available, SRA, sequence read archive*

*Source:* [*https://www.ncbi.nlm.nih.gov/*](https://www.ncbi.nlm.nih.gov/)

***Supplementary Table S4*** *Details of molecular markers, genetic map and QTLs available in small millets.*

| **Component** | **Barnyard millet^a^** | **Finger millet^b^** | **Foxtail millet^c^** | **Kodo millet^d^** | **Little millet^e^** | **Proso millet^f^** | **References** |
| --- | --- | --- | --- | --- | --- | --- | --- |
| **Marker type*** |  |  |  |  |  |  |  |
| SSR | 5  3  23  78 | 82  46  87  101 | 269  143  172  78  21,294  788  10,598 | 11  19  17 | NA | 548 709 | ^a^ Danquah et al., 2002, Nozawa et al., 2006, Lee at al., 2014, Chen et al., 2017,  ^b^ Dida et al., 2007, Babu et al., 2014a, Ramakrishnan et al., 2016.  Gimode et al., 2016  ^c^ Jia et al., 2009, Zhao et al., 2012, Gupta et al., 2013,  Pandey et al., 2013, Zhang et al., 2014, Fang et al., 2016  ^d^ Cidade et al., 2009, Cidade et al., 2010, Cidade et al., 2013  ^f^ Rajput et al., 2016, Rajput and Santra, 2016 |
| EST-SSRs | 22  51  30 | 132  545  58  74  56 | 447  17,117 | NA | NA | 313 | ^a^ Babu et al., 2017, Manimekalai et al., 2018, Murukarthick et al., 2019  ^b^ Babu et al., 2014d, Babu et al., 2014c, Babu et al., 2014b,  Nirgude et al., 2014,  ^c^ Obidiegwu et al., 2013, Kumari et al., 2013  ^f^ Cho et al., 2010, Hou et al., 2017 |
| SNPs | 19,033  49,179  3  2 | 92  23,000 | 16,77,516 25,84,083 33,579 9,968  845  787  751  992 | 3461 | 2245 | 1882 833 | ^a^ Wallace et al., 2015, Chen et al., 2017, Ishikawa et al., 2013, Babu et al.,  2017  ^b^ Gimode et al., 2016,  ^c^ Bai et al., 2013, Wang et al., 2017, Jia et al., 2013, Zhang et al., 2012,  Bennetzen et al., 2012,  ^d^ Johnson et al., 2019  ^e^ Johnson et al., 2019  ^f^ Johnson et al., 2019, Zou et al., 2019 |
| InDels | NA | NA | 55,348  24  4 | NA | NA | 1 | ^c^ Bai et al., 2013, Yoshitsu et al., 2017, Sun et al., 2019  ^f^ Hunt et al., 2010 |
| **Molecular map** |  |  |  |  |  |  |  |
| Genetic map | NA | 2 | 7 | NA | NA | 1 | ^c^ Jia et al., 2009, Bennetzen et al., 2012, Zhang et al., 2012, Qie et al.,  2014, Sato et al., 2013, Fang et al., 2016, Wang et al., 2017 |
| Physical map | NA | NA | 2 | NA | NA | NA | ^c^ Kumari et al., 2013, Zhang et al., 2014 |
| Haplotype map | NA | NA | 2 | NA | NA | NA | ^c^ Jia et al., 2013 |
| **QTLs/genes** |  |  |  |  |  |  |  |
| Morpho-agronomic traits | NA | 5  13  9 | 7  59  29  9  18  3  61  521  5  57  11 | NA | NA | 18 | ^b^ Ramakrishnan et al., 2016. Lule et al., 2018, Sharma et al., 2018  ^c^ Jaiswal et al., 2019, ,Zhang et al., 2017, Fang et al., 2016, Gupta et al., 2014,  Qie et al., 2014, Jia et al., 2013, Wang et al., 2013, Sato et al.,  2013, Mauro-Herrera et al., 2013, Wang et al., 2017  ^f^ Zou et al., 2019 |
| Abiotic/biotic stress related traits | NA | 4  2 | 12  18 | NA | NA | NA | ^b^ Ramakrishnan et al., 2017,  Ramakrishnan et al., 2016  ^c^ Mauro‐Herrera et al., 2013, Veeranagamallaiah et al., 2009, Mishra et al.,  2012, Lata and Prasad, 2012, Qie et al., 2014 |
| Nutritional traits | NA | 3  9 | 2 | NA | NA | NA | ^b^ Babu et al., 2014b, Kumar et al., 2015  ^c^ Bai et.al., 2013 |

*Note: *markers reported here are excluding of cross species markers, NA, not available*
